# Preparation of a Ti_0.7_W_0.3_O_2_/TiO_2_ nanocomposite interfacial photocatalyst and its photocatalytic degradation of phenol pollutants in wastewater†

**DOI:** 10.1039/c9na00478e

**Published:** 2019-12-12

**Authors:** Zemin Dong, Rendan Zhou, Leyan Xiong, Han Li, Qiang Liu, Longzhen Zheng, Zanru Guo, Zhaoxiang Deng

**Affiliations:** Department of Chemistry and Chemical Engineering, East China Jiao Tong University Nanchang 330013 P. R. China xly12@ecjtu.edu.cn zemin1987d@139.com; JiangXi Institute for Veterinary Drug and Feedstuffs Control Nanchang 330096 PR China; Analysis and Testing Center, Nan Chang University Nanchang 330047 P. R. China; CAS Key Laboratory of Crust-Mantle Materials and Environments, School of Earth and Space Sciences, University of Science and Technology of China Hefei Anhui 230026 P. R. China; Department of Chemistry, University of Science and Technology of China Hefei Anhui 230026 P. R. China

## Abstract

A Ti_0.7_W_0.3_O_2_/TiO_2_ nanocomposite interfacial photocatalyst was designed and prepared for the photocatalytic degradation of phenol pollutants in wastewater. The detailed properties of the Ti_0.7_W_0.3_O_2_/TiO_2_ nanocomposite interface (NCI) were analyzed by XRD, SEM, EDX, DRS, UPS and XPS technologies, showing that anatase TiO_2_ nanospheres (NSs) were uniformly dispersed on the surface of rutile Ti_0.7_W_0.3_O_2_ nanoparticles (NPs) and formed the nanocomposite interface. The DRS and UPS results of 5 wt% Ti_0.7_W_0.3_O_2_/TiO_2_ NCI indicated a greatly broadened light response range with a wavelength shorter than 527 nm and a shorter band gap energy of 2.37 eV. The conduction band of TiO_2_ NSs, Ti_0.7_W_0.3_O_2_ NPs and 5 wt% Ti_0.7_W_0.3_O_2_/TiO_2_ NCI were measured based on the results of the valence band and band gap energy obtained *via* XPS and DRS, and then the energy level diagram of Ti_0.7_W_0.3_O_2_/TiO_2_ NCI was proposed. The photocatalytic degradation of phenol at Ti_0.7_W_0.3_O_2_/TiO_2_ NCI with different loading ratios of Ti_0.7_W_0.3_O_2_ NPs was investigated under optimum conditions (*i.e.*, pH of 4.5, catalyst dosage of 0.45 g L^−1^ and phenol initial concentration of 95 ppm) under the illumination of ultraviolet visible light. Also, 5 wt% Ti_0.7_W_0.3_O_2_/TiO_2_ NCI exhibited the highest photocatalytic activity, with the initial rate constant (*k*) calculated as 0.09111 min^−1^. After recycling six times, Ti_0.7_W_0.3_O_2_/TiO_2_ NCI showed good stability and recyclability. The involvement of superoxide radicals in the initial reaction at Ti_0.7_W_0.3_O_2_/TiO_2_ NCI was evidenced by the use of a terephthalic acid (TA) fluorescent probe. Besides, UV-Vis spectroscopy, UHPLC-MS and GC-MS technologies were used to analyze the main intermediates in the photocatalytic degradation of phenol. The probable photocatalytic degradation mechanism of phenol at Ti_0.7_W_0.3_O_2_/TiO_2_ NCI was also proposed.

## Abstract 2

The Ti_0.7_W_0.3_O_2_/TiO_2_ nanocomposite interfacial photocatalyst was designed and prepared for the photocatalytic degradation of phenol pollutants in wastewater.

## Graphical abstract

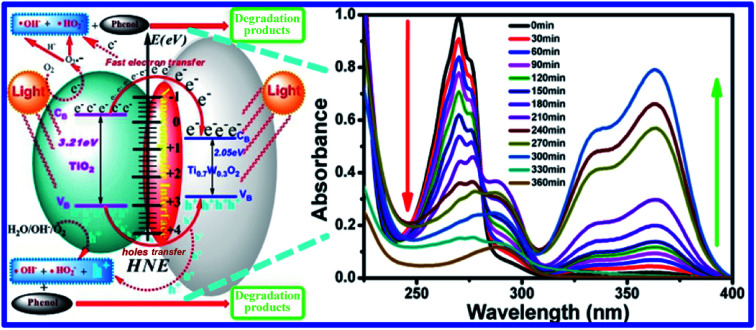

## 1. Introduction

Environment pollution has become a worldwide problem. Organic wastewater is one of the most typical pollution issues posing a serious threat to the health of humans and has consequently received extensive attention recently.^1–3^ Over the last several years, the utilization of direct solar light has become a much greener approach for energy generation as well as for environmental clean-up. Therefore, the development and design of a UV-Vis active photocatalyst for direct sunlight harvesting has drawn broad interdisciplinary attention and much research fascination. The photocatalytic decomposition of organic contaminants is one of the most promising techniques for wastewater treatment and purification.

Among oxide semiconductor photocatalysts, TiO_2_ nanomaterials have been studied and applied widely in photocatalysis for their high photocatalytic activity, stability, nontoxicity and low cost. A variety of functional TiO_2_ nanomaterials have been synthetized, such as nanoparticles (NPs),^4–9^ nanotubes (NTs),^10,11^ nanowires (NWs),^12–15^ nanocrystal films^16–20^ and nanotube arrays,^21–25^ which have been widely used in solar energy storage and utilization, photodegradation of pollutants and noble metal recycling.

However, the total quantum efficiency of TiO_2_ is very low,^26–28^ which has limited the potential value of actual production and application of TiO_2_ nanomaterials. A lot of studies have been done to address the drawbacks mentioned above, with noble metal deposition considered as one of the most effective and promising solutions. Pt is one such representative noble metal, which has been widely used to improve the performance of TiO_2_ nanomaterials in wastewater treatment and air purification with a superior performance. Wang^29^ successfully synthesised a Pt/TiO_2_ NW photocatalyst. The recombination rate of electrons and holes was reduced greatly for Pt NPs, resulting in good conductivity. Pt NPs are superior electron acceptors on the photocatalyst surface and enable the timely transfer of electrons. Emilio *et al.*^30^ observed an increase in the lifetime of electrons by Pt modification on the TiO_2_ surface due to the better separation of charge carriers caused by the Schottky barrier between Pt and TiO_2_. As expected, this helped to enhance the photocatalytic efficiency of TiO_2_.^5–9,11,14,15,19,20,22,25,31–33^ However, noble metals are scarce and particularly expensive, which may limit their large-scale application. Thus, novel relatively economical photocatalysts are highly desirable.

In the present paper, a Ti_0.7_W_0.3_O_2_/TiO_2_ NCI was synthesized *via* a sol–gel and combustion technique, and was shown to possess several positive aspects, such as good stability, good visible light response range and effectively decreased recombination of charge carriers by a fast photogenerated electron transfer. The photocatalytic activity of the Ti_0.7_W_0.3_O_2_/TiO_2_ NCI was investigated for the degradation of phenol under simulated solar light illumination, and it showed higher photocatalytic activity. Furthermore, the main intermediates and mechanism for the photocatalytic degradation of phenol at the Ti_0.7_W_0.3_O_2_/TiO_2_ NCI were also analyzed and discussed. This type of a photocatalyst may find application in low concentration organic wastewater clean-up.

## 2 Experiment section

### 2.1 Materials and reagents

Titanium tetrachloride (99.9%), titanium trichloride (99.9%), WCl_6_ (99.9%), hexachloroplatinic acid (99.9%), carbolic acid (99.9%), HPLC-grade methanol, HPLC-grade acetonitrile and other reagents were all purchased from Shanghai Chemical Reagent Factory. Pure TiO_2_ samples were commercial Degussa P-25 (55 m^2^ g^−1^). Doubly distilled water was used throughout this study. All the chemicals were of analytical grade and were used without further purification.

### 2.2 Synthesis of the TiO_2_NSs

The pure anatase TiO_2_ NSs was prepared *via* a hydrothermal method.^34^ First, 6 mM NaOH particles were added to 40 mL absolute ethyl alcohol and stirred for 10 min. Then, 2 mL titanium trichloride solution was added to the above NaOH solution drop-wise under vigorous stirring. After 10 min, the mixed solution was transferred to a 50 mL Teflon-lined autoclave and heated at 150 °C for 18 h. After cooling, the precipitate collected through centrifugation was rinsed with distilled water and pure ethanol several times until there were no residual ions left. Then, the products were calcined at 400 °C for 2 h after being dried at 80 °C.

### 2.3 Synthesis of the Ti_0.7_W_0.3_O_2_ NPs

The Ti_0.7_W_0.3_O_2_ NPs were prepared by a modified sol–gel technique. First, 4 mM of WCl_6_ powder was added to 10 mL of absolute ethyl alcohol with stirring for 10 min. Then, 1 mL of titanium tetrachloride solution was added to 5 mL of distilled water drop-wise under vigorous stirring. The above solutions were mixed together under air-free conditions. Then, the above mixed solution was stirred by mechanical stirring under 40 °C constant temperature water for about 24 h to get a baby blue gel. The product was poured into a drying oven at 100 °C for 12 h. The dry power was added to a 50 mL Teflon-lined autoclave with a moderate amount of alcohol and heated at 180 °C for 8.5 h, and then the precipitate was dried and ground. Finally, the obtained powder was reduced at 1300 °C in a H_2_ atmosphere for 4 h to obtain a light tan powder. At this point, the W^6+^ was fully reduced to W^4+^.^35,36^

### 2.4 Synthesis of the Ti_0.7_W_0.3_O_2_/TiO_2_ NCI

The Ti_0.7_W_0.3_O_2_/TiO_2_ NCI was prepared *via* a simple method. First, 6 mM NaOH were added to 40 mL absolute ethyl alcohol and stirred for 10 min. Then, 2 mL titanium trichloride solution was added to the above NaOH solution drop-wise under vigorous stirring accompanied with a certain quality of pure rutile Ti_0.7_W_0.3_O_2_ NPs.^34^ After 30 min, the mixed solution was transferred to a 50 mL Teflon-lined autoclave and heated at 180 °C for 8.5 h, and then the precipitate obtained was dried and ground. Then, the obtained powders were calcined at 400 °C in a N_2_ atmosphere for 2 h. All referenced Ti_0.7_W_0.3_O_2_/TiO_2_ NCI samples were also prepared by the method described above and used for the photoactivity tests.

### 2.5 Synthesis of the Pt/TiO_2_ nanocomposites

The synthesis of the Pt/TiO_2_ nanocomposites and the characterization results are described in the ESI (Section 1, Fig. S1–S4†).

Meanwhile, the detailed information on the characterization, photocatalytic test and analysis of the intermediates in the photocatalytic degradation of phenol are shown in ESI (Section 2†).

## 3 Results and discussion

### 3.1 Characterization

**Fig. 1:**
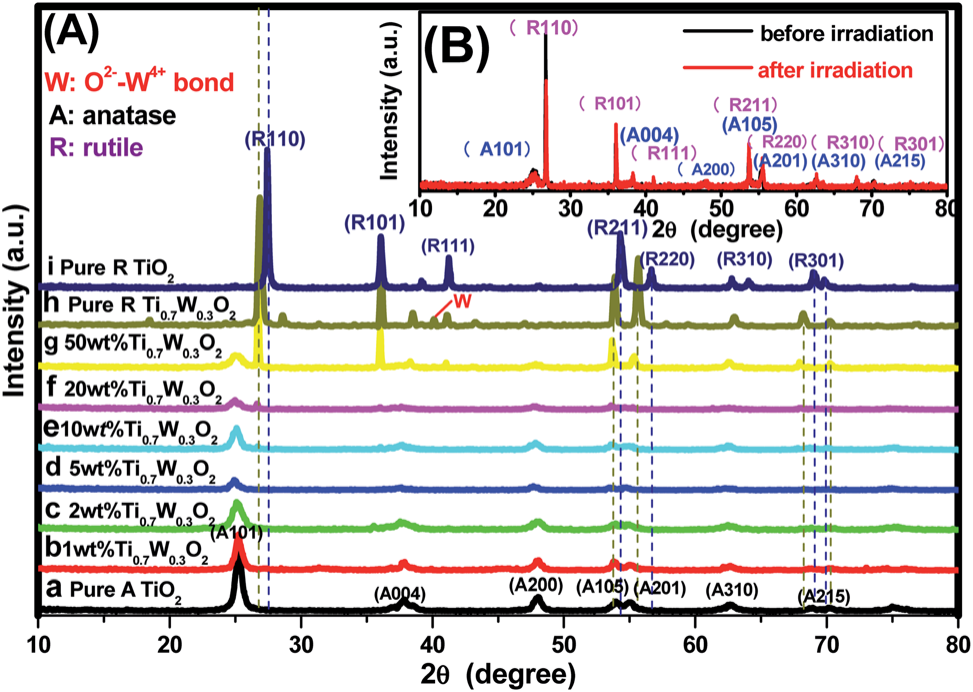
PXRD patterns of the tested samples: (A) (a) pure anatase TiO_2_, Ti_0.7_W_0.3_O_2_/TiO_2_ NCI of (b) 1 wt%, (c) 2 wt%, (d) 5 wt%, (e) 10 wt%, (f) 20 wt%, (g) 50 wt%, (h) pure rutile Ti_0.7_W_0.3_O_2_, (i) pure rutile TiO_2_; (B) 50 wt% Ti_0.7_W_0.3_O_2_/TiO_2_ NCI before and after six cycles of irradiation, respectively.

PXRD analysis was carried out to investigate the impact of Ti_0.7_W_0.3_O_2_ modification on the phase structure and on the chemical composition of the TiO_2_ NSs, as these have a great influence on the photocatalytic activity. The PXRD patterns of the prepared samples are depicted in Fig. 1(A). According to previous studies, the photocatalytic property of anatase TiO_2_ is generally considered to be superior, attributed to a higher density of localized states, consequent surface-adsorbed hydroxyl radicals and slower charge-carrier recombination.^37–39^ All diffraction peaks of the samples (a and i) could be indexed to the International Centre for Diffraction data of pure anatase TiO_2_ (JCPDS no. 21-1272) and rutile TiO_2_ (JCPDS no. 21-1276), respectively. The crystallite sizes were calculated by Scherrer's formula given in eqn (1).

1 *D* = *Kλ*/*β* cos *θ*

where λ is the wavelength of the Cu-Kα used, *β* is the full width at half-maximum of the diffraction peak, *K* is a shape factor (0.94) and *θ* is the angle of diffraction. The average crystalline size calculated from the major diffraction peak (101) of anatase TiO_2_ NSs was about 149.1 nm.^34^ The average crystalline size calculated from the major diffraction peak (110) of rutile Ti_0.7_W_0.3_O_2_ NPs was about 1077 nm.^36^

The average particle size and distribution of the anatase TiO_2_ NSs and Ti_0.7_W_0.3_O_2_ NPs were obtained using a laser particle size analyzer and are shown in ESI (Section 3, Fig. S6†). The average particle size and distribution of anatase TiO_2_ NSs were determined to be about 287 nm, which was larger than the average crystalline size calculated from the major diffraction peak (101) in the XRD analysis. The average particle size and distribution of Ti_0.7_W_0.3_O_2_ NPs were determined to be about 1189 nm, which was consistent with the result calculated from the major diffraction peak (110) in the XRD analysis of the rutile Ti_0.7_W_0.3_O_2_ NPs. The possible reasons for the deviation were as follows. On the one hand, some TiO_2_ NSs reunite after high temperature calcination. On the other hand, the principles of the two kinds of detection methods were different, whereby the results of the XRD analysis were estimated using an empirical formula, whereas the laser particle size analyzer detection needed the samples to be dispersed in water, and the dispersion of the TiO_2_ NSs was not very good and they were prone to reunion. This might lead to an increase in the error of the result.

Obviously, some diffraction peaks of Ti_0.7_W_0.3_O_2_ NPs (h) were slightly shifted due to doping with W^4+^ compared with pure rutile TiO_2_ (i). This phenomenon indicated an expansion of the *a*-axis and a contraction of the *c*-axis due to W–W pairing in the doped compound, which has also been observed in WO_2_/TiO_2_ nanocomposites.^40–45^ Meanwhile, the diffraction peaks for O^2−^–W^4+^ were very weak, demonstrating the low levels of W^4+^.

The PXRD patterns of Ti_0.7_W_0.3_O_2_/TiO_2_ NCI loaded with 1 wt%, 2 wt%, 5 wt%, 10 wt%, 20 wt% and 50 wt% Ti_0.7_W_0.3_O_2_ NPs are shown in Fig. 1(A)(b–g). All the samples were identical with the pure anatase and rutile phase after calcination at 400 °C_,_ respectively. None of the diffraction peaks were changed significantly after deposition, which indicated that the Ti_0.7_W_0.3_O_2_ NPs did not affect the phase structure and chemical composition of the TiO_2_ NSs. However, further observation showed that the diffraction peaks corresponding to TiO_2_ NSs exhibited relatively weaker peak intensities and broader diffraction peak widths. It could be inferred from this that the average crystallite size was slightly decreased by Ti_0.7_W_0.3_O_2_ NPs modification, indicating that the Ti_0.7_W_0.3_O_2_ NPs have a negative effect on the grain growth of TiO_2_ NSs. This is because the Ti_0.7_W_0.3_O_2_ NPs restrained the crystal growth in the solids by providing dissimilar boundaries and hindered the mass transportation, thus resulting in smaller crystallite sizes.^46^ Meanwhile, no diffraction peaks of Ti_0.7_W_0.3_O_2_ NPs were observed up to 10 wt% (e), indicating that the TiO_2_ NSs were uniformly dispersed on the surface of the Ti_0.7_W_0.3_O_2_ NPs.

The superimposed PXRD patterns for the Ti_0.7_W_0.3_O_2_/TiO_2_ NCI before and after six cycles of irradiation are shown in Fig. 1(B). It is obvious that the two PXRD patterns almost overlap, which indicates that the stability of the Ti_0.7_W_0.3_O_2_/TiO_2_ NCI was encouraging, with less decomposition, thus accounting for the higher photocatalytic activity. A feeble and relatively weaker peak intensity was also revealed for the loss of a certain amount of photocatalyst during the experiment.

**Fig. 2:**
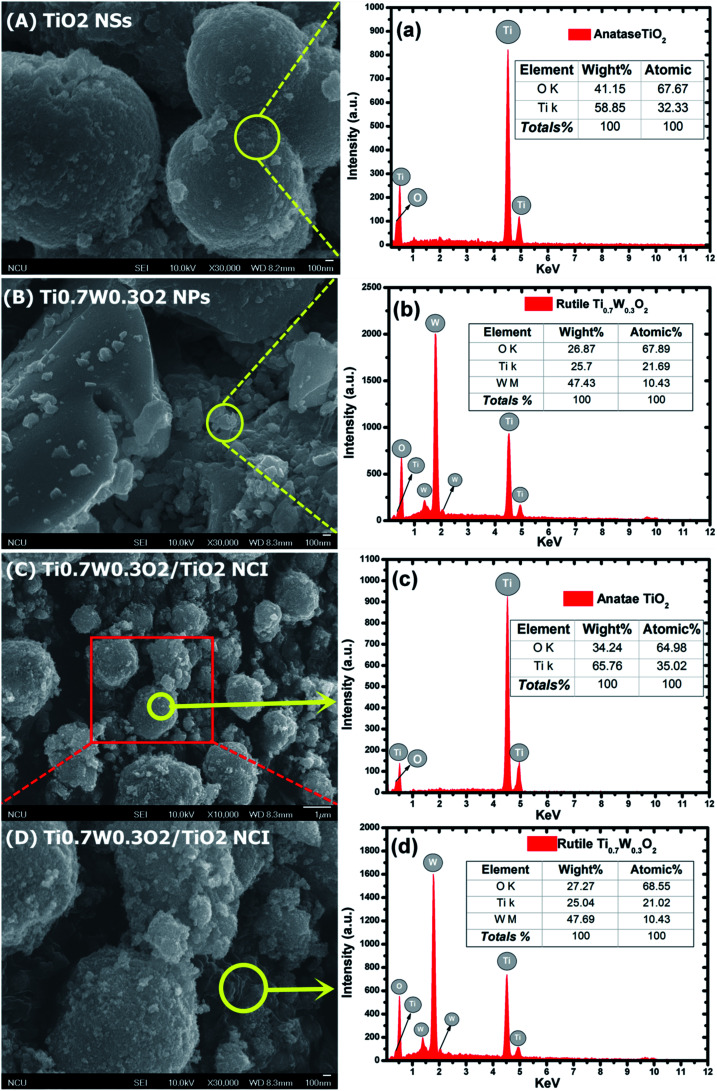
SEM images of: (A) anatase TiO_2_, (B) Ti_0.7_W_0.3_O_2_ and (C)/(D) 5 wt% Ti_0.7_W_0.3_O_2_/TiO_2_ NCI. The corresponding EDX test results of: (a) anatase TiO_2_, (b) Ti_0.7_W_0.3_O_2_ and (c) and (d) 5 wt% Ti_0.7_W_0.3_O_2_/TiO_2_ NCI, respectively.

The detailed morphological features of the Ti_0.7_W_0.3_O_2_/TiO_2_ NCI were characterized by SEM technology and are shown in Fig. 2(A–D). The pure anatase TiO_2_ was present as nanospheres, with a uniform size distribution as shown in Fig. 2(A). Fig. 2(B) shows the morphology of the pure rutile Ti_0.7_W_0.3_O_2_ NPs, which were irregular and schistose particles with a smooth surface and highly dense quality. Fig. 2(C) and (D) show that the TiO_2_ NSs were uniformly dispersed on the surface of the Ti_0.7_W_0.3_O_2_ NPs and formed the Ti_0.7_W_0.3_O_2_/TiO_2_ NCI.

The elemental composition of the synthesis of the samples was confirmed by EDX spectra. In Fig. 2(a and b), the anatase TiO_2_ revealed major peaks of Ti and O, and the rutile Ti_0.7_W_0.3_O_2_ NPs revealed major peaks of Ti, W and O. Fig. 2(c and d) evidence the presence of Ti, W and O elements for the Ti_0.7_W_0.3_O_2_/TiO_2_ NCI, indicating the high purity, and we can easily distinguish between the two components in the composite photocatalyst.^47–49^

Furthermore, the atomic% of Ti/the atomic% of O of anatase TiO_2_ was measured as 0.48, which was close to the mol ratio of Ti/O (0.50) in TiO_2_. The atomic% of Ti/the atomic% of W of rutile Ti_0.7_W_0.3_O_2_ NPs was measured as 2.08, which was close to the mol ratio of Ti/W (2.33) in Ti_0.7_W_0.3_O_2_. The EDX results for each synthesized sample were a little different with the theoretical mol ratio of elements as EDX merely involved a local analysis of the entire surface of samples, and so these represented acceptable errors.^50–52^

However, the atomic% of Ti/the atomic% of O of anatase TiO_2_ and the atomic% of Ti/the atomic% of W of rutile Ti_0.7_W_0.3_O_2_ NPs in Ti_0.7_W_0.3_O_2_/TiO_2_ NCI were measured to be 0.54 and 2.02, respectively. The error of the results had thus increased, which might be due to the interaction between the two nanomaterials.

**Fig. 3:**
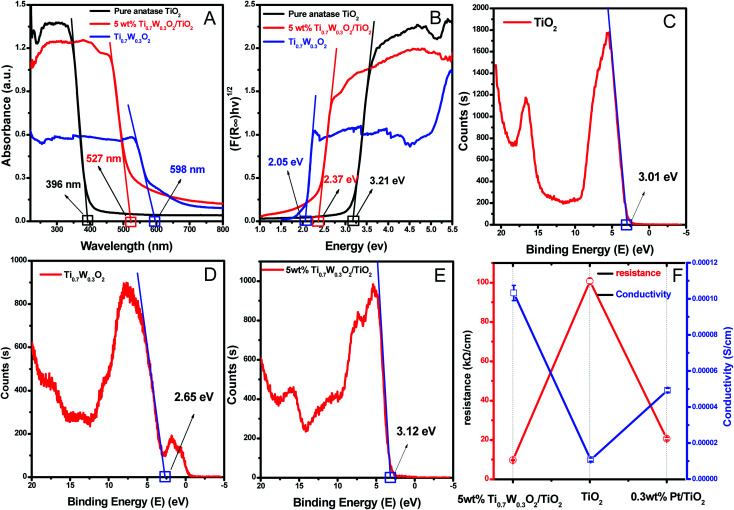
(A) DRS and (B) plots of the transformed Kubelka–Munk function *versus* the energy of absorbed light for the pure anatase TiO_2_, Ti_0.7_W_0.3_O_2_ and 5 wt% Ti_0.7_W_0.3_O_2_/TiO_2_ NCI. The XPS valence band scan spectra of: (C) pure anatase TiO_2_ NSs, (D) rutile Ti_0.7_W_0.3_O_2_ and (E) 5 wt% Ti_0.7_W_0.3_O_2_/TiO_2_ NCI. (F) The volume resistivity and conductivity of pure anatase TiO_2_, 0.3 wt% Pt/TiO_2_ and 5 wt% Ti_0.7_W_0.3_O_2_/TiO_2_ NCI.

A UV-Vis spectrometer was used to record diffuse reflectance spectra in the range 200–800 nm. Fig. 3 (A) shows the DRS of pure TiO_2_ NSs, pure rutile Ti_0.7_W_0.3_O_2_ and 5 wt% Ti_0.7_W_0.3_O_2_/TiO_2_ NCI. The band gap values of the synthesized photocatalysts were calculated by plotting (*F*(*R*∞)*hv*)^1/2^*versus* the photo energy and the plot is shown in Fig. 3 (B). The pure TiO_2_ NSs and the pure rutile Ti_0.7_W_0.3_O_2_ demonstrated a photoabsorption modification ability for the UV light region with wavelength shorter than 396 and 598 nm, corresponding to band gap energies of 3.21 and 2.05 eV, respectively. The pure rutile Ti_0.7_W_0.3_O_2_ had a shorter band gap energy due to W^4+^ doped into the lattice of TiO_2_. When the pure rutile Ti_0.7_W_0.3_O_2_ was irradiated, conduction band electrons (e_cb_^−^) were generated and quickly spread to the valence band due to the shorter band gap energy, which might make the semiconductor have higher conductivity.^53^ The volume resistivity and conductivity of pure anatase TiO_2_, 0.3 wt% Pt/TiO_2_ and 5 wt% Ti_0.7_W_0.3_O_2_/TiO_2_ are shown in Fig. 3(F). The volume resistivity of pure anatase TiO_2_ was over 10 times that of 5 wt% Ti_0.7_W_0.3_O_2_/TiO_2_. The volume resistivity of 0.3 wt% Pt/TiO_2_ was over 2 times that of 5 wt% Ti_0.7_W_0.3_O_2_/TiO_2_. It was thus indicated that the conductivity of pure anatase TiO_2_ could be improved greatly by modifying the Ti_0.7_W_0.3_O_2_ NPs, and that the performance of Ti_0.7_W_0.3_O_2_ NPs was superior to that of Pt NPs, which could fully prove the above conjecture.

Meanwhile, the 5 wt% Ti_0.7_W_0.3_O_2_/TiO_2_ NCI was extended to the visible absorbance region with a wavelength shorter than 527 nm and had a shorter band gap energy of 2.37 eV. The above results were fully proved by ultraviolet photoemission spectroscopy (UPS) and the results are shown in ESI (Section 4, Fig. S7†). The band gap energies of the pure TiO_2_ NSs and 5 wt% Ti_0.7_W_0.3_O_2_/TiO_2_ NCI were 3.38 and 2.43 eV, respectively, which were slightly larger than that from the DRS. The reasons for the deviation may be due, on the one hand, to the detection depth of UPS technology, which was 10 atoms, while on the other hand, the carbon pollution signal would be higher for the solid powder sample.

In short, Ti_0.7_W_0.3_O_2_ NPs with higher conductivity can cause fast electron transfer and effectively restrain the recombination of e_cb_^−^–h_vb_^+^ pairs in Ti_0.7_W_0.3_O_2_/TiO_2_ NCI, which can diffuse to the surface and react with pollutants and produce more superoxide radical anions (˙O_2_^−^)/˙OH^−^. Furthermore, the Ti_0.7_W_0.3_O_2_/TiO_2_ NCI could also improve the utilization of visible light, which might account for the higher photocatalytic activity.

The XPS valence band scan spectra of pure anatase TiO_2_ NSs, rutile Ti_0.7_W_0.3_O_2_ and 5 wt% Ti_0.7_W_0.3_O_2_/TiO_2_ NCI are shown in Fig. 3(C–E). The valence bands of TiO_2_ NSs, rutile Ti_0.7_W_0.3_O_2_ and 5 wt% Ti_0.7_W_0.3_O_2_/TiO_2_ NCI were 3.01, 2.65 and 2.83 eV, respectively. According to the band gap energy result from DRS, the conduction bands were measured as −0.20, 0.60 and 0.46 eV, and the energy level diagram of Ti_0.7_W_0.3_O_2_/TiO_2_ NCI could be proposed and is shown in ESI (Section 4, Fig. S8†).

**Fig. 4:**
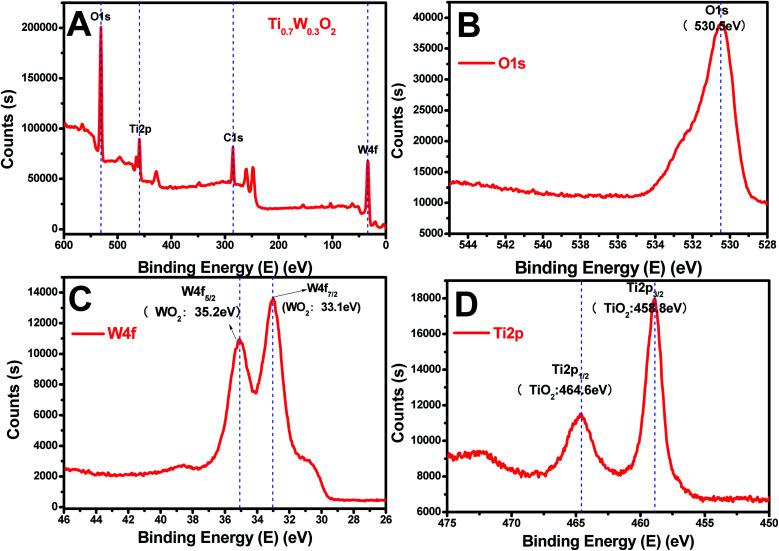
XPS survey scan spectra of: (A) rutile Ti_0.7_W_0.3_O_2_, and XPS narrow scans of: (B) O 1s, (C) W 4f and (D) Ti 2p of the rutile Ti_0.7_W_0.3_O_2_.

The rutile Ti_0.7_W_0.3_O_2_ was further characterized by XPS, as seen in Fig. 4(A–D), to illustrate the structural features and composition. The XPS survey scan spectrum of rutile Ti_0.7_W_0.3_O_2_ is shown in Fig. 4(A). The relative concentrations of Ti and W of Ti_0.7_W_0.3_O_2_ were determined by the respective XPS peak areas and atomic sensitivity factors and *n*_Ti_/*n*_W_ was measured as 2.13, which is close to the mol ratio of Ti/W (2.33) in Ti_0.7_W_0.3_O_2_ ^54^ and consistent with the EDX result.

The XPS narrow scan spectra of Ti 2p, W 4f and O 1s of the rutile Ti_0.7_W_0.3_O_2_ are shown in Fig. 4(B–D). The Ti 2p_1/2_ and Ti 2p_3/2_ peaks of Ti_0.7_W_0.3_O_2_ were located at 464.6 and 458.8 eV and assigned to TiO_2_, respectively. The W 4f_5/2_ and W 4f_7/2_ peaks were located at 34.2 and 33.1 eV, and assigned to WO_2_, proving that the W^6+^ was fully reduced to W^4+^ in Ti_0.7_W_0.3_O_2_.

### 3.2 Photocatalysis

#### 3.2.1 Optimal photocatalytic conditions (pH, initial phenol concentration, photocatalyst dosage)

**Fig. 5:**
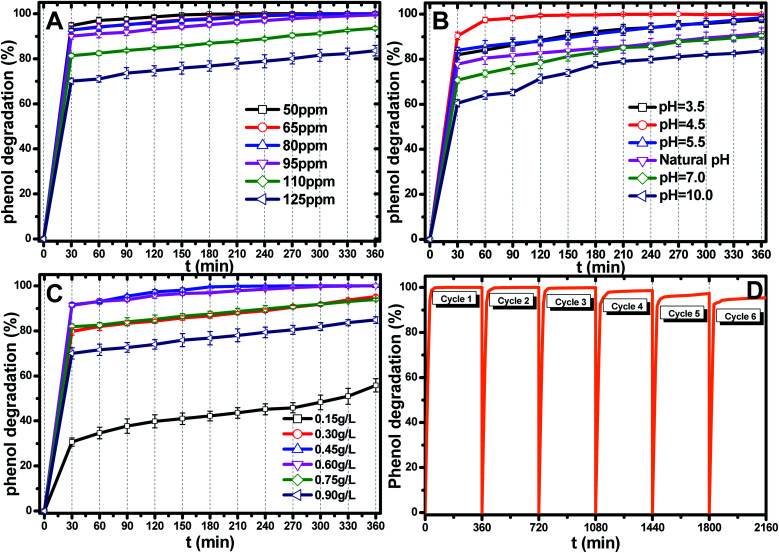
(A) Effect of the initial pH value on phenol photocatalytic degradation; catalyst dosage: 0.45 g L^−1^, phenol concentration: 95 ppm. (B) Effect of the initial catalyst dosage on phenol photocatalytic degradation; pH: 4.5, phenol concentration: 95 ppm. (C) Effect of the initial phenol concentration on phenol photocatalytic degradation; catalyst dosage: 0.45 g L^−1^, pH: 4.5. (D) Recyclability of the Ti_0.7_W_0.3_O_2_/TiO_2_ NCI in the phenol photodegradation process under visible light illumination through six cycles at initial concentration: 95 ppm, pH: 4.5, catalyst dosage: 0.45 g L^−1^.

In order to find the optimal initial pH value of the solution, the photocatalytic degradation of phenol was carried out at a pH of 3.5–10.0, catalyst dosage of 0.45 g L^−1^, irradiation time of 360 min and phenol concentration of 95 ppm and the results are shown in Fig. 5(A). Obviously, the Ti_0.7_W_0.3_O_2_/TiO_2_ NCI showed the highest photocatalytic activity under pH 4.5, indicating that phenol photodegradation in the acidic solution was higher than that in natural solution, neutral solution and alkaline solution, which was consistent with the research conclusions of Khataee^55^ and Kim.^56^ They believed that the oxidation ability of the hydroxyl radical (˙OH^−^) under acidic conditions was higher than in alkaline solution. The formation of HCO_3_^−^ and CO_3_^2−^ in alkaline solution would interfere in the reaction between pollutants and ˙OH^−^, resulting in reducing its oxidation potential and leading to a lower photocatalytic activity. Meanwhile, with the appearance of HCO_3_^−^ and CO_3_^2−^, the low adsorption of negatively charged system components resulted in a lower production of superoxide radical anions (˙O_2_^−^) and hence a lower oxidation ability. However, the phenol photodegradation at pH 3.5 was also lower than that at pH 4.5. This may be due to the change in the Ti_0.7_W_0.3_O_2_/TiO_2_ NCI structure under a too acidic environment. Herein, the results revealed that the optimal initial pH value was 4.5.

The most appropriate initial phenol concentration was investigated with the initial concentration ranging from 50–125 ppm and the results are shown in Fig. 5(B). Obviously, the complete photodegradation time of phenol increased with the increase in the initial concentration from 50–95 ppm, and the photodegradation of phenol at 95 ppm could be just finished with 360 min irradiation. The photocatalytic efficiencies of 110 ppm and 125 ppm phenol were 94.3% and 86.3% after 360 min irradiation, respectively. Further increases decreased the photocatalytic efficiency, indicating that there was an optimum value. The reasonable explanations for this are as follows: first, too many phenol molecules and its intermediates would also absorb a part of the irradiation and limit the light absorption capability of the photocatalysts. Second, excessive amounts of phenol molecules and its intermediates also deactivate more active sites and reduce the light penetration to active sites situated on the surfaces of Ti_0.7_W_0.3_O_2_/TiO_2_ NCI. The above two disadvantages also result in a lower production of superoxide radical anions (˙O_2_^−^)/˙OH^−^ and ultimately a lower oxidation ability.^57^

The effect of the Ti_0.7_W_0.3_O_2_/TiO_2_ NCI dosage was investigated by varying the dosage from 0.15 g L^−1^ to 0.90 g L^−1^ and the results are shown in Fig. 5(C). When raising the Ti_0.7_W_0.3_O_2_/TiO_2_ NCI dosage from 0.15 g L^−1^ to 0.60 g L^−1^, the phenol photocatalytic efficiency increased from 58.7% to 100% as more active sites were available, increasing the response surface area and leading to a greater production of ˙O_2_^−^/˙OH^−^. However, further increasing, the dosage to 0.90 g L^−1^ decreased the photocatalytic efficiency. According to the literature,^58–62^ the reasons for this might be due to the following aspects: on the one hand, an excessive dosage of photocatalysts would result in lower solution transparency, light scattering and interception and the prevention of the light induction of some catalysts particles. On the other hand, too many photocatalysts particles would prevent the effective collisions between phenol molecules and a variety of free radicals. Moreover, the pore volume and available surface area of the photocatalysts would also be diminished with excessive dosage, resulting in a lower photocatalytic activity.

The stability and recyclability of all heterogeneous photocatalysts are critically important for application in wastewater treatment plants. The stability and recyclability of Ti_0.7_W_0.3_O_2_/TiO_2_ NCI were investigated in a batch reactor under pH 4.5, a catalyst dosage of 0.45 g L^−1^ and phenol concentration of 95 ppm. After each experiment, the used photocatalyst was collected from the suspension turbid solution and washed with 50% ethanol solution to remove residue phenol and other photodegradation products on the photocatalysts surface. Then, the wet photocatalyst was dried at 105 °C for 4 h. This sequence was repeated six times and the phenol photodegradation efficiency of each cycle recorded and the results are shown in Fig. 5(D). After six recycles, the photocatalytic degradation efficiency of Ti_0.7_W_0.3_O_2_/TiO_2_ NCI was reduced from 100% to 94.5%, indicating that the Ti_0.7_W_0.3_O_2_/TiO_2_ NCI showed high photocatalytic activity with good stability and recyclability. The reduction could be explained by a loss of photocatalyst during the washing process, which was consistent with the PXRD results.

#### 3.2.2 Comparison of the phenol photocatalytic degradation efficiency of P-25, Pt/TiO_2_ and Ti_0.7_W_0.3_O_2_/TiO_2_ NCI

The photocatalytic activity of Ti_0.7_W_0.3_O_2_/TiO_2_ NCI was examined by monitoring phenol photocatalytic degradation under UV-visible light illumination and then compared with P-25 and Pt/TiO_2_. All experiments were carried out under pH 4.5, a catalyst dosage of 0.45 g L^−1^ and phenol initial concentration of 95 ppm with the highest photocatalytic activity. The UV-Vis spectra of the photocatalytic degradation of phenol by P-25, Pt/TiO_2_ and Ti_0.7_W_0.3_O_2_/TiO_2_ NCI are shown in ESI (Section 5, Fig. S9†).

**Fig. 6:**
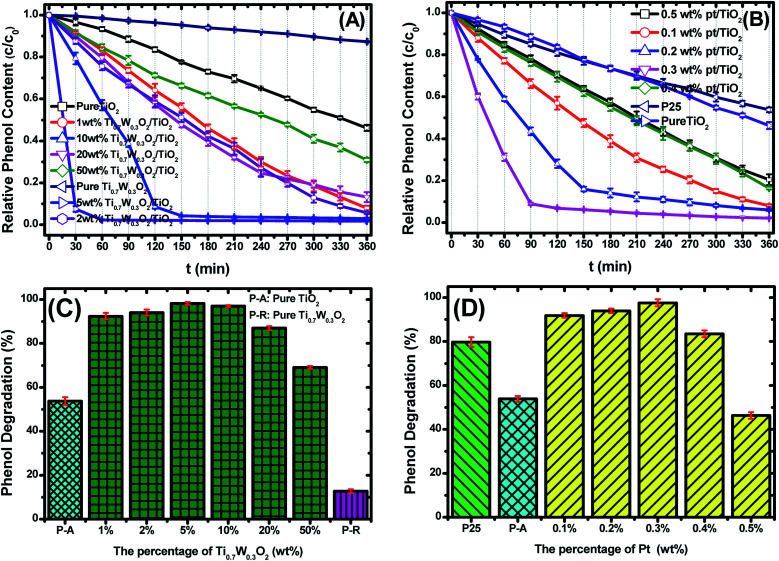
Photocatalytic degradation of phenol over: (A) Ti_0.7_W_0.3_O_2_/TiO_2_ NCI, (B) P-25 and Pt/TiO_2_, *c* = concentration, *c*_0_ = initial concentration. The phenol photocatalytic degradation rates of: (C) Ti_0.7_W_0.3_O_2_/TiO_2_ NCI, (D) P-25 and Pt/TiO_2_ after 360 min irradiation.

Fig. 6(A and B) present the phenol photocatalytic degradation by P-25, Pt/TiO_2_ and Ti_0.7_W_0.3_O_2_/TiO_2_ NCI, showing the differences in the phenol degradation activity with the varying loading rates of Ti_0.7_W_0.3_O_2_/TiO_2_ and Pt/TiO_2_. The phenol degradation rate of Ti_0.7_W_0.3_O_2_/TiO_2_ NCI increased with the loading value of Ti_0.7_W_0.3_O_2_ up to 5 wt%; however, a further increase would decrease the photocatalytic activity, indicating that there was an optimum loading value. The optimum value had a close relationship with the dispersion and particle sizes of Ti_0.7_W_0.3_O_2_ NPs. Meanwhile, one could easily find that the phenol degradation rate of 5 wt% Ti_0.7_W_0.3_O_2_/TiO_2_ NCI was always higher than that of 0.3 wt% Pt/TiO_2_ at any synchronous irradiation time, revealing its higher photocatalytic activity.

Fig. 6(C and D) compare the phenol photocatalytic degradation rates of P-25, Pt/TiO_2_ and Ti_0.7_W_0.3_O_2_/TiO_2_ NCI after 360 min irradiation. The phenol photocatalytic degradation rate after various intervals of time was estimated using the following eqn (2).

2 Phenol photocatalytic degradation rate (%) = (*c*_*t*=0_ − *c*_*t*_)/*c*_*t*=0_ × 100%

where *c*_*t*=0_ is the initial concentration of phenol and *c*_*t*_ is the concentration of phenol obtained after various intervals of time (*t*). From the experimental study, it was observed that 5 wt% Ti_0.7_W_0.3_O_2_/TiO_2_ NCI showed higher photocatalytical activity. The phenol photocatalytic degradation trend was: 5 wt% > 10 wt% > 2 wt% > 1 wt% > 20 wt% > 50 wt% > pure TiO_2_ > pure Ti_0.7_W_0.3_O_2_. Here, 98.7% of the phenol was photodegraded by 5 wt% Ti_0.7_W_0.3_O_2_/TiO_2_ NCI after 50 min irradiation, indicating a higher photocatalytic activity. However, only about 47.23% and 90.79% of the phenol was photodegraded for P-25 and 0.3 wt% Pt/TiO_2_ after 360 min irradiation. It was indicated that the Ti_0.7_W_0.3_O_2_ NPs may be much superior to Pt NPs for modifying the photocatalytic performance of TiO_2_ nanomaterial.

#### 3.2.3 Kinetic study of the phenol photocatalytic degradation

**Fig. 7:**
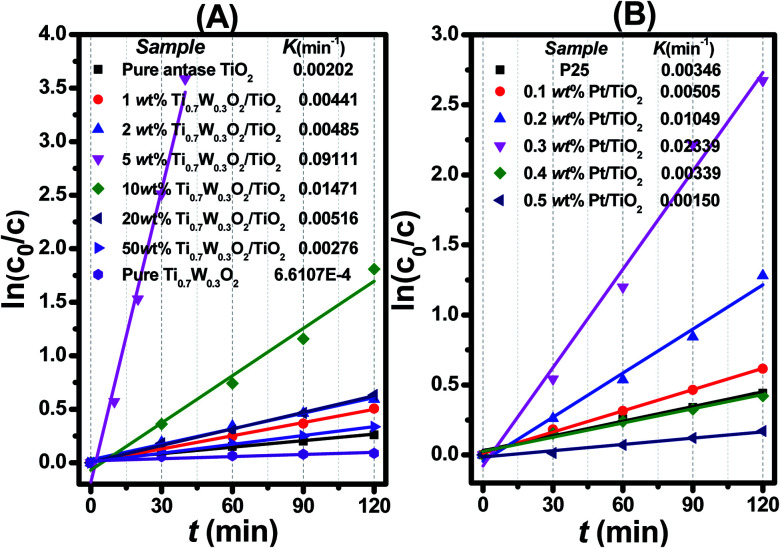
Dependence of In (*c*_0_/*c*) on the irradiation time (*t*) for: (A) Ti_0.7_W_0.3_O_2_/TiO_2_ NCI, (B) P25 and Pt/TiO_2_.

Additionally, kinetic analysis of phenol degradation was performed for a better comparison of the photocatalytic efficiency of the different photocatalysts. The dependence of ln(*c*_0_/*c*) on the irradiation time (*t*) in P-25, Pt/TiO_2_ and Ti_0.7_W_0.3_O_2_/TiO_2_ is shown in Fig. 7. It was indicated that the initial photodegradation of phenol followed a quasi-first-order-type kinetics, as evidenced by the linear relationship between ln(*c*_0_/*c*) and the time (*t*). Actually, *c*_0_ is the initial concentration of phenol, and *c* is the concentration of phenol after irradiation for time (*t*).

The initial rate constant (*k*) for phenol photocatalytic degradation in P-25 was calculated as 0.00346 min^−1^, while the initial rate constant (*k*) for phenol photocatalytic degradation in 5 wt% Ti_0.7_W_0.3_O_2_/TiO_2_ and 0.3 wt% Pt/TiO_2_ was about 26 and 6.8 times that of P-25, respectively. This indicated that the photocatalytic activity of TiO_2_ was enormously improved with the proper amount of loading Ti_0.7_W_0.3_O_2_ and Pt NPs. Furthermore, the initial rate constant (*k*) for 5 wt% Ti_0.7_W_0.3_O_2_/TiO_2_ was over 3.9 times that in 0.3 wt% Pt/TiO_2_, illustrating that the as-prepared Ti_0.7_W_0.3_O_2_ NPs may be much superior to Pt NPs in embellishing the photocatalytic properties of TiO_2_ nanomaterials and could even replace them.

Herein, to better assess the photocatalytic activity of the synthesized Ti_0.7_W_0.3_O_2_/TiO_2_ NCI, we compared our results with the photodegradation of phenol reported in previous studies, as shown in Table 1. The Ti_0.7_W_0.3_O_2_/TiO_2_ NCI showed several advantages in the photocatalytic performance, photocatalytically degrading the most amount of phenol with the least irradiation time and catalyst dosage. The initial rate constant (*k*) of the Ti_0.7_W_0.3_O_2_/TiO_2_ NCI was over 2.6 times that of SnS_2_/TiO_2_ nanocomposite catalyst, which showed a higher photocatalytic activity than the other catalysts. Therefore, it was concluded that the Ti_0.7_W_0.3_O_2_/TiO_2_ NCI was one of the most efficient catalysts for the photocatalytic degradation of phenol under the selected experimental parameters.

**Table 1:** Comparison of the photocatalytic degradation of phenol in this study with the results reported in the open literature

Catalyst	Concentration (mg L^−1^)/volume (mL) of phenol	Catalyst amount (g L^−1^)	Degradation (%)	Irradiation time (min)	Initial rate constant (*k*) (min^−1^)	Reference
Pt–ZnO	15	—	>95	540	—	63
ZnO	50/200	1.0	69.75	480	0.0150	64
GO/TiO_2_	14/100	1.48	100	180	—	65
RGO/TiO_2_	50/1700	—	96	180	0.0154	66
MWCNT/TiO_2_	50/800	1.0	96	300	0.0074	67
Fe/S/TiO_2_	20/60	1.0	99.4	600	—	68
CNT/Ce–TiO_2_	50/500	0.4	95	180	0.0012	69
BiPO_4_	50/100	0.5	100	240	0.0370	70
Co/Pd/BiVO_4_	18.4/100	0.8	90	180	0.0130	71
ZnO/TiO_2_	60/250	0.6	100	160	0.0124	72
TiO_2−*x*_B_*x*_	94/50	6	97	240	0.0084	73
BiMnO_4_	20/100	1.0	90	480	0.0049	74
Fe(iii)–TiO_2_	100/1500	0.5	93.8	210	0.0190	75
TiO_2_/Ag/C	20/100	1.0	95	60	—	76
N–TiO_2_@CS	9.4/40	2.5	90	180	—	77
V_2_O_5_/N,S–TiO_2_	100/20	1.0	88	240	—	78
Pt/TiO_2_	—	—	87.7	180	—	79
TiO_2_–Fe_2_O_3_–graphene	5/100	1.5	—	150	0.01415	80
SnS_2_/TiO_2_	10/100	0.5	—	150	0.03595	81
***Ti***_***0.7***_ ***W***_***0.3***_ ***O***_***2***_ ***/TiO***_***2***_ ***NCI***	95/1000	0.45	98.7	50	0.09111	***This study***

#### 3.2.4 Analysis of the intermediates in the photocatalytic degradation of phenol

It is well known that phenol decomposition could follow different complicated multistage pathways, following various by-products. It has also been demonstrated that two types of oxidizing species, namely hydroxyl radicals and positive holes, are involved in oxygenated aqueous TiO_2_ suspensions.^79–84^ Ilisz *et al.*^85^ found that phenol was degraded into various by-products (such as hydroquinone, catechol and other ring-opened compounds) with different oxidizing agents under UV-Vis irradiation in the presence of different electron scavengers. Catechol, hydroquinone and 2,4-hexadiendioic acid were found during phenol degradation at mesoporous TiO_2−*x*_B_*x*_ by Xiong *et al.*^73^

The UV-Vis absorption spectra from the photodegradation of phenol over P-25, Pt/TiO_2_ and Ti_0.7_W_0.3_O_2_/TiO_2_ are compared in ESI (Section 6, Fig. S10†). At P-25, besides the characteristic absorption bands at 270 nm of phenol, a new absorption band at 289 nm appeared, which might be attributed to the ring-retaining compounds.^29,59,86,87^ However, besides the two absorption bands at 270 and 289 nm, there were two new absorption bands at 247 and 257 nm for Pt/TiO_2_, and another two new absorption bands at 333 and 363 nm for Ti_0.7_W_0.3_O_2_/TiO_2_, which might be attributed to ring-opened produciits.^87,88^ It can be concluded that the phenol photodegradation pathway over Ti_0.7_W_0.3_O_2_/TiO_2_ NCI was partially different from that over P-25 and Pt/TiO_2_.

The aqueous solutions of phenol degradation over P25, Pt/TiO_2_ and Ti_0.7_W_0.3_O_2_/TiO_2_ were detected by UV-Vis spectrometry, UHPLC-MS and GC-MS. Then, the main intermediates were analyzed and inferred by the molecular ions and mass fragment peaks present and from library data. The LC chromatograms, UV-Vis spectrograms and mass spectra from HPLC-MS are shown in ESI (Section 7, Fig. S11a–k†). The GC chromatograms and mass spectra from GC-MS of the intermediates are shown in ESI (Section 8, Fig. S12 and S13†). The analytical results and possible structures of each intermediate are shown in ESI (Section 9, Table S1 and Fig. S14†).

In short, five and six kinds of intermediates were identified in the aqueous suspension of P-25 and Pt/TiO_2_, respectively. Six kinds of intermediates were found in the aqueous suspension of Ti_0.7_W_0.3_O_2_/TiO_2_. The further degradation of all the intermediates might include oxidative hydroxylation and oxidative decarboxylation products, *etc.* from several reaction pathways operating simultaneously.

#### 3.2.5 Determination of the superoxide radicals using a terephthalic acid (TA) fluorescent probe

**Fig. 8:**
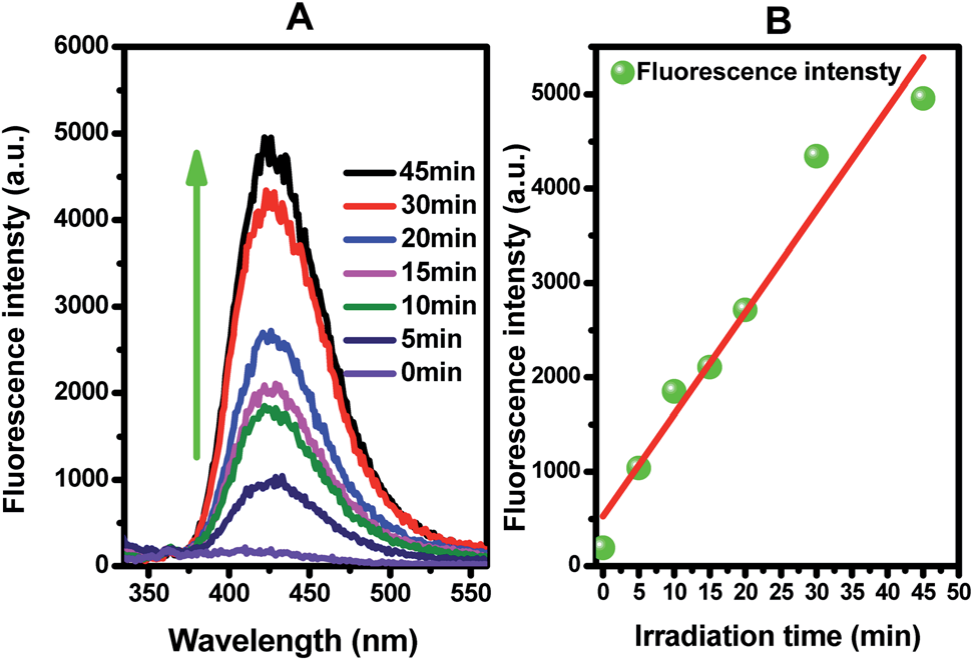
(A) Fluorescence spectral changes observed during the illumination of Ti_0.7_W_0.3_O_2_/TiO_2_ NCI in 0.01 M NaOH and 3 mM terephthalic acid solution (under 315 nm excitation); (B) plots showing the induced fluorescence intensities (425 nm) against light illumination time for terephthalic acid.

Superoxide radicals can react with TA and generate 2-hydroxyterephthalic acid (TAOH), which emits fluorescence at around 425 nm on the excitation of its own 315 nm absorption band.^89–93^Fig. 8(A) shows the fluorescence spectra observed for the supernatant solution of the Ti_0.7_W_0.3_O_2_/TiO_2_ NCI suspension containing 3 mM TA irradiated for various irradiation times. Since the observed fluorescence spectra were identical to that of TAOH, it was concluded that TAOH is generated from TA by the reaction with superoxide radical anion (˙O^2−^) and hydroxyl radical (˙OH^−^), where superoxide radicals are generated in Ti_0.7_W_0.3_O_2_/TiO_2_ NCI suspension and involved in the radical reaction mechanism.

Figure 8(B) presents the fluorescence intensity as a function of the duration of irradiation. The fluorescence intensity increased linearly with the irradiation time, showing that the formation superoxide radical follows the quasi-first-order-type kinetics, as evidenced by the linear relationship between the concentration of superoxide radical and irradiation time within a certain range.

#### 3.2.6 Mechanism of the photocatalytic degradation of phenol

It has been proposed that TiO_2_ modified with different materials may result in different photodegradation intermediates, indicating different decomposition mechanisms.^81–83^ The radical reaction and holes reaction mechanism of phenol degradation with mesoporous TiO_2−*x*_B_*x*_ was proposed by Xiong *et al.*^73^ Liu *et al.*^94^ well studied the mechanisms of phenol degradation over TiO_2_ 3D microspheres and proposed three kinds of photodegradation pathways: phenol was transformed into dihydroxybenzene, benzoquinone and 4,4′-dihydroxybiphenyl first, and then transformed into maleic anhydride, which was further photodegraded to CO_2_ and H_2_O, finally. Their conclusion was consistent with the results of other research groups of anatase TiO_2_ photocatalytic materials.^95–97^

**Fig. 9:**
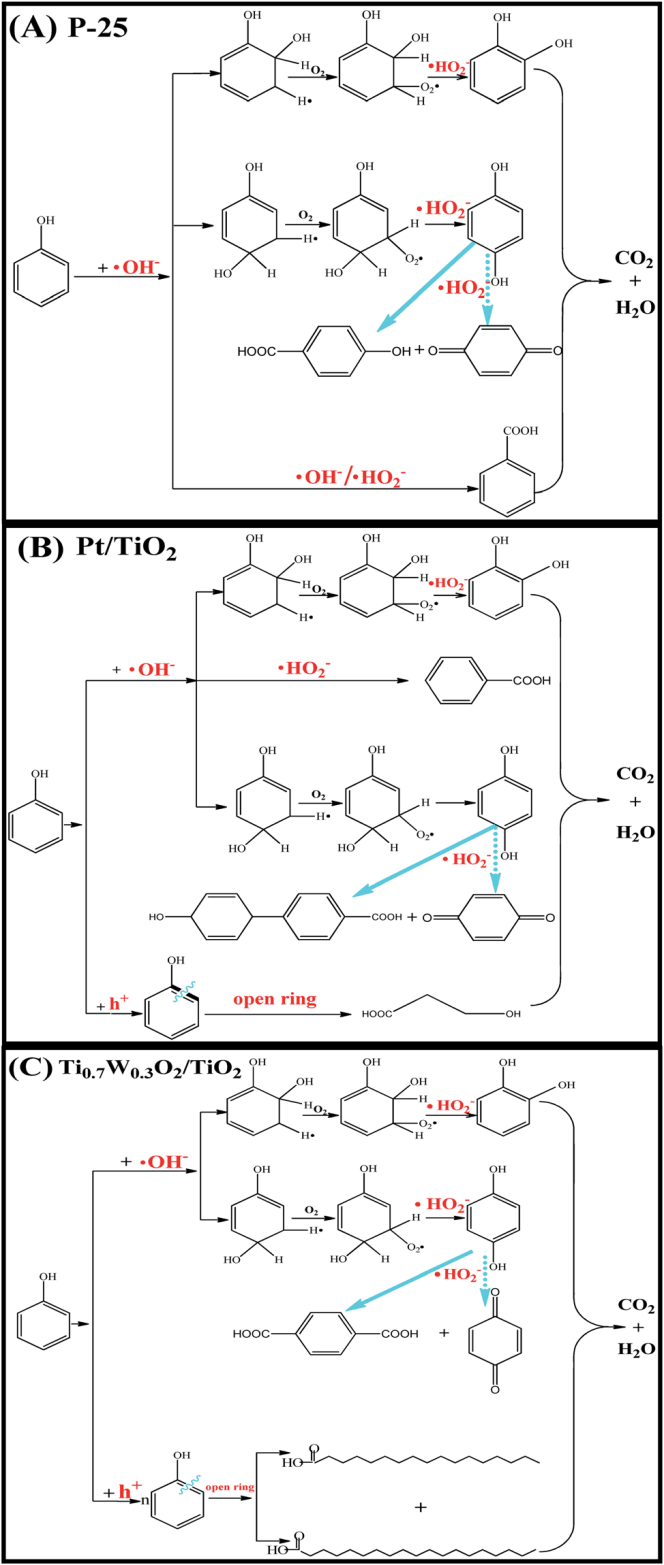
Proposed photocatalytic degradation process of phenol over: (A) P-25, (B) Pt/TiO_2_ and (C) Ti_0.7_W_0.3_O_2_/TiO_2_ NCI.

Therefore, based on the present experimental data and the referenced studies,^29,59,91,94–97^ the different intermediates of P-25, Pt/TiO_2_ and Ti_0.7_W_0.3_O_2_/TiO_2_ indicate the different phenol degradation processes, as clearly illustrated in Fig. 9.

In addition, we believe that the photocatalytic degradation of phenol over P-25 follows a radical reaction mechanism. The photocatalytic degradation of phenol over Pt/TiO_2_ and Ti_0.7_W_0.3_O_2_/TiO_2_ follows both a radical reaction mechanism and holes reaction mechanism, which proceed in parallel.

**Scheme 1:**
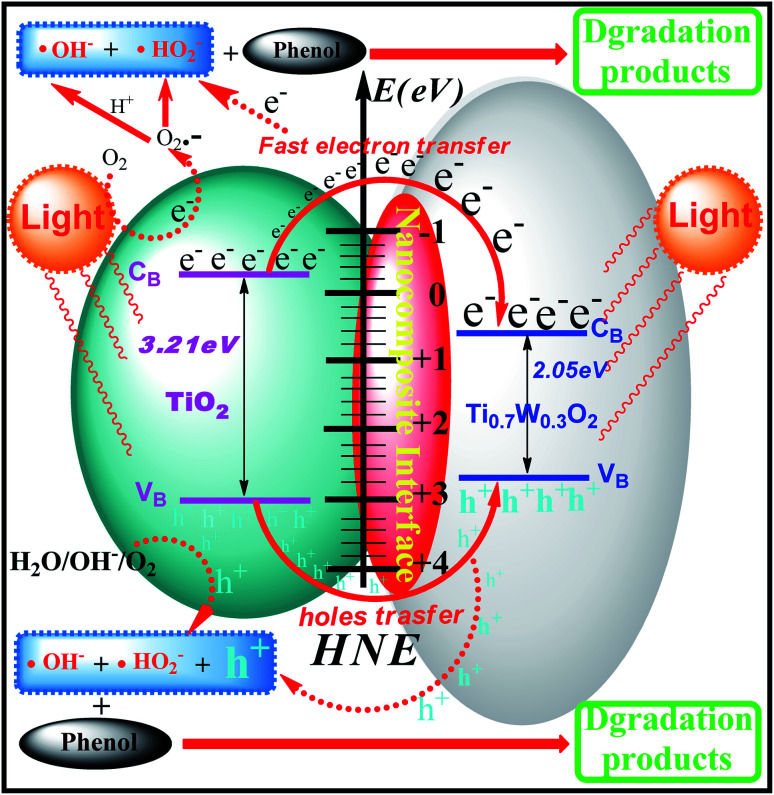
Proposed phenol photocatalytic degradation mechanism at the Ti_0.7_W_0.3_O_2_/TiO_2_ NCI.

The phenol photocatalytic degradation mechanism involves initial reactions at the Ti_0.7_W_0.3_O_2_/TiO_2_ NCI, as shown in Scheme 1. It was well established that conduction band electrons (e_cb_^−^) and valence band holes (h_vb_^+^) were generated when the suspension was irradiated. A Schottky barrier might be formed at the NCI between TiO_2_ NSs and Ti_0.7_W_0.3_O_2_ NPs, leading to a greater formation of h_vb_^+^ and e_cb_^−^ and an enhanced photocatalytic activity.^59^ Ti_0.7_W_0.3_O_2_ NPs with high conductivity might cause fast electron transfer and effectively restrain the recombination of e_cb_^−^–h_vb_^+^ pairs in the bulk catalyst, which can then diffuse to the surface and react with pollutants.^54^ Hydroxyl radical (˙OH^−^) formation occurred at the Ti_0.7_W_0.3_O_2_/TiO_2_ NCI by h_vb_^+^ trapping absorbed hydroxyl and hydration molecules (OH^−^/H_2_O). Meanwhile, e_cb_^−^ could react with molecular oxygen (O_2_) adsorbed at the NCI and produce superoxide radical anion (˙O_2_^−^). Acidic conditions could generate a higher affinity towards unpaired e_cb_^−^ of NCI, leading to the formation of more hydroxyl radicals (˙HO_2_^−^).^98,99^ The h_vb_^+^ could also oxidize pollutants directly. These might account for the extraordinary photocatalytic activity of Ti_0.7_W_0.3_O_2_/TiO_2_ NCI.

## 4. Conclusions

In summary, Ti_0.7_W_0.3_O_2_/TiO_2_ nanocomposite interfacial photocatalysts with loading of different weight ratios of Ti_0.7_W_0.3_O_2_NPs were designed and synthesized for the photocatalytic degradation of phenol in wastewater under the illumination of ultraviolet visible light. The optimum photocatalytic degradation of phenol conditions were pH 4.5, a catalyst dosage of 0.45 g L^−1^ and phenol initial concentration of 95 ppm. The 5 wt% Ti_0.7_W_0.3_O_2_ NPs was the best loading level, and the initial rate constant (*k*) for 5 wt% Ti_0.7_W_0.3_O_2_/TiO_2_ NCI was over 3.9 times that in 0.3 wt% Pt/TiO_2_. The valence bands of TiO_2_ NSs, Ti_0.7_W_0.3_O_2_ NPs and 5 wt% Ti_0.7_W_0.3_O_2_/TiO_2_ NCI were 3.01, 2.65 and 2.83 eV, the band gap energies were 3.21, 2.05 and 2.37 eV, respectively. Then the conduction bands of the above three materials were measured to be −0.20, 0.60 and 0.46 eV. Ti_0.7_W_0.3_O_2_/TiO_2_ NCI showed a greatly broadened light response range, shorter band gap energy and good stability and recyclability. A Schottky barrier was formed at the NCI between TiO_2_ NSs and Ti_0.7_W_0.3_O_2_ NPs, leading to a higher formation of h_vb_^+^ and e_cb_^−^, and the Ti_0.7_W_0.3_O_2_ NPs could quickly transfer e_cb_^−^ and effectively restrain the recombination of e_cb_^−^–h_vb_^+^ pairs for high conductivity. A large number of superoxide radicals were generated in the suspension in the photocatalytic degradation of phenol by the Ti_0.7_W_0.3_O_2_/TiO_2_ NCI, which enhanced the photocatalytic activity. Besides, five and six kinds of intermediates were identified in the suspension of P-25 and Pt/TiO_2_, respectively, and six kinds of intermediates were found in the suspension of Ti_0.7_W_0.3_O_2_/TiO_2_. The photocatalytic degradation of phenol over P-25 followed a radical reaction mechanism. The photocatalytic degradation of phenol over Pt/TiO_2_ and Ti_0.7_W_0.3_O_2_/TiO_2_ followed both a radical reaction mechanism and hole reaction mechanism, which proceeded in parallel.

## Conflicts of interest

The authors declare no competing financial interest.

## Supplementary Material

NA-002-C9NA00478E-s001
