# Supplementary material for: Preparation of a Ti0.7W0.3O2/TiO2 nanocomposite interfacial photocatalyst and its photocatalytic degradation of phenol pollutants in wastewater
Source: Nanoscale Adv. 2019 Dec 12;2(1):425–37. doi: 10.1039/c9na00478e (PMC9418697; doi:10.1039/c9na00478e)
Supplement: NA-002-C9NA00478E-s001 [file NA-002-C9NA00478E-s001.pdf]

Electronic Supplementary Material (ESI) for Nanoscale Advances.
This journal is © The Royal Society of Chemistry 2019

Electronic Supplementary Information

Preparation of $Ti_{0.7}W_{0.3}O_2/TiO_2$ nanocomposite interfacial photocatalyst and its photocatalytic degradation of phenol pollutant in wastewater

Zemin Dong[a,b], Rendan Zhou[c], Leyan Xiong*[,b], Li Han[d], Qiang Liu[b], Longzhen Zheng[b] and Zhaoxiang Deng[e]

NO.of Supporting Pages: 24.

NO.of Supporting Figures: 14.

NO.of Supporting Tables: 1.

a.JiangXi Institute for Veterinary Drug and Feedstuffs Control, Nanchang 330096, PR China

b.Department of Chemistry and Chemical Engineering, East China Jiao Tong University, Nanchang 330013, P.R. China

c.Analysis and Testing Center, Nan Chang University, Nanchang 330047, P.R. China

d.CAS Key Laboratory of Crust-Mantle Materials and Environments, School of Earth and Space Sciences, University of Science and Technology of China, Hefei, Anhui 230026, P.R. China

e.Department of Chemistry, University of Science and Technology of China, Hefei, Anhui 230026, P.R. China

Section 1. The synthesis and characterization of Pt/TiO_2 nanocomposite

A. Synthesis of Pt/TiO_2 nanocomposite

The deposition of Pt on TiO_2 was carried out via a simple photochemical reduction method. 0.3 g TiO_2 nanospheres was dissolved in 20 ml distilled water, and treated ultrasonically 10 min for dispersion. Then 2 ml 2-propanol and a given amount of H_2PtCl_6 were added and stirred 30 min for the adsorption on TiO_2 nanospheres surface. The mixed solution was saturated in a test tube sealed with a rubber septum and irradiated by high-pressure mercury lamp under vigorous stirring. Under the irradiation, the light-generated h^+_{TiO2} oxidize 2-propanol to inorganic products and at the same time the light-generated e^-_{TiO2} reduce the platinum ions (Pt^{4+}) to the metallic form (Pt^0). The transformation of the Pt^{4+} to Pt^0 state could be inferred from the change in the solution color from slightly yellow to light gray. The irradiation lasted for 3 h to complete reaction. There was no loss of Pt in the preparation process. So, the Pt doping content was 0.1, 0.2, 0.3, 0.4 and 0.5 wt %, respectively.

B. The PXRD analysis of Pt/TiO_2 nanocomposite

The PXRD patterns of anatase TiO_2 and 0.3wt% Pt/TiO_2 photocatalyst were depicted in Figure S1. All diffraction peaks of TiO_2 NSs in Figure S1 (A) and (B) could be indexed to international center for diffraction data of pure anatase TiO_2 (JCPDS No.21-1272),respectively. Meanwhile, the diffraction peaks at 39.2°,45.682° and 66.5° were ascribed to (111), (200) and (220) planes of Pt. It clearly indicated that the synthesis of Pt/TiO_2 photocatalyst was achieved.

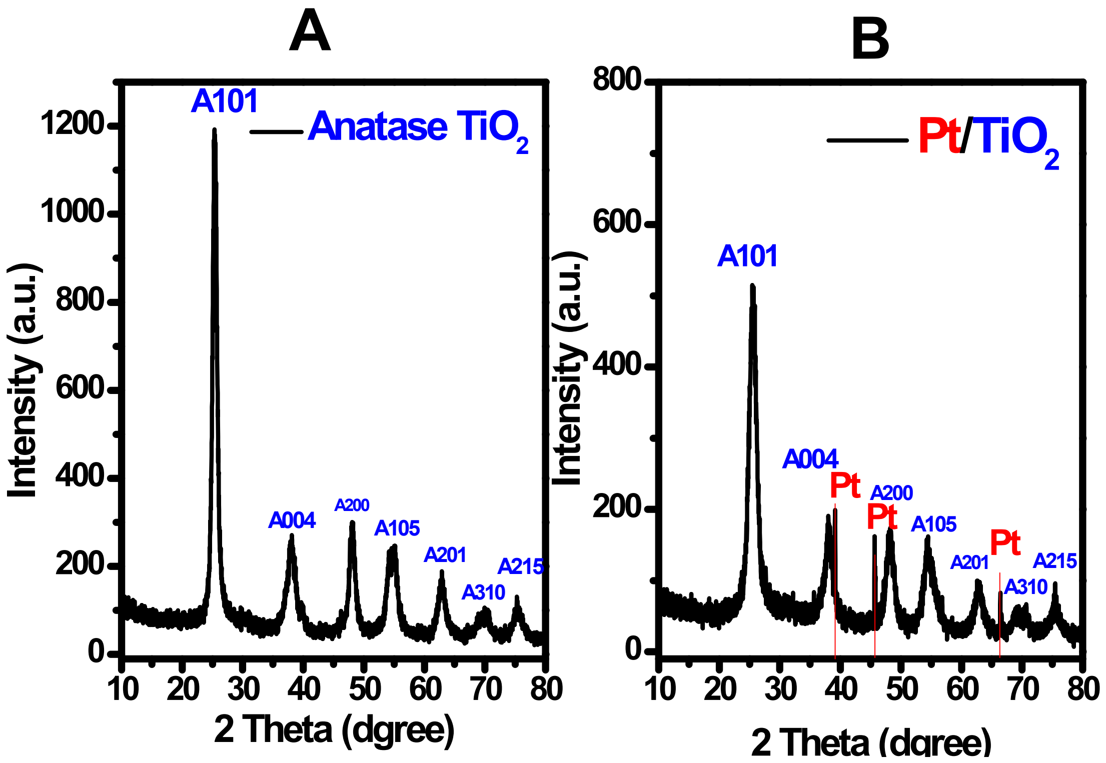

Figure S1 PXRD patterns of samples: (A) pure anatase TiO_2 and (B) 0.3 wt % Pt/TiO_2 photocatalyst, respectively.

C. The DRS analysis of Pt/TiO_2 nanocomposite

The UV-Vis spectrometer was used to record diffuse reflectance spectra of anatase TiO_2 and 0.3 wt% Pt/TiO_2 photocatalyst in the range 200~800 nm. Figure S2 (A) showed the DRS of anatase TiO_2 and 0.3 wt% Pt/TiO_2 photocatalyst. The band gap values of the synthesized photocatalysts were calculated by plots of $(F(R\infty)hv)^{1/2}$ versus photo energy and shown in Figure S2 (B).The anatase TiO_2 presented the photo-absorption modify ability of the UV light region with a wavelength shorter than 396 nm, corresponding to the band gap energy of 3.21 eV, which was in agreement with previous work. But the 0.3 wt% Pt/TiO_2 photocatalyst extended to the visible absorbance region with a wavelength shorter than 449 nm and shorter band gap energy of 3.07 eV. The Pt Nps can improve the utilization of the visible light and shorter band gap energy accounting for the higher photoactivity than pure anatase TiO_2.

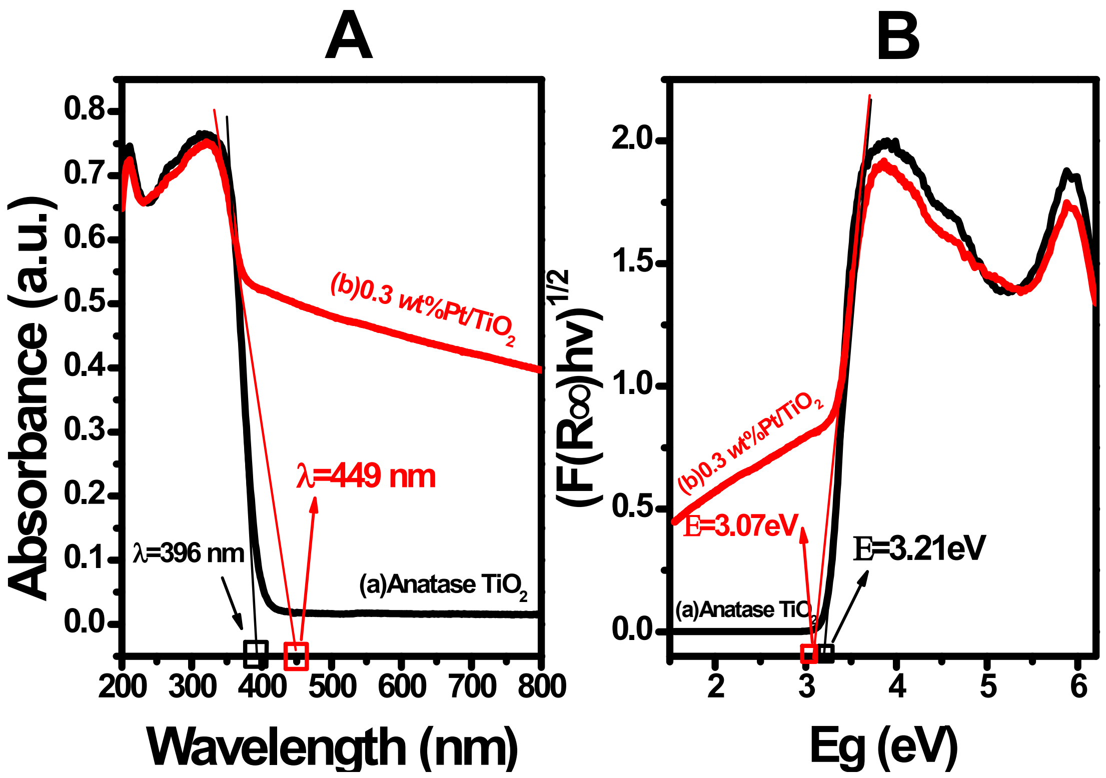

Figure S2 (A) DRS and (B) plots of transformed Kubelka–Munk function versus the energy of absorbed light for the pure anatase TiO_2 and 0.3 wt % Pt/TiO_2 photocatalyst, respectively.

D. The XPS valence band scan spectrum analysis of Pt/TiO_2 nanocomposite

The XPS valence band scan spectrum result of anatase TiO_2 and 0.3 wt% Pt/TiO_2 photocatalyst was shown in Figure S3. The valence band of 0.3 wt% Pt/TiO_2 photocatalyst was 2.59 eV, which was lower than that of pure anatase TiO_2 NSs. After H_2PtCl_6 was reduced, uniform Pt NPs were deposited onto the surface of TiO_2 NSs. According to the results of DRS, the band gap energy of the 0.3 wt% Pt/TiO_2 was 3.07 eV, and the conduction band of 0.3 wt% Pt/TiO_2 can be measured to be -0.48 eV. It indicated that the 0.3 wt% Pt/TiO_2 has shorter band gap energy and fast electron transfer and higher photoactivity.

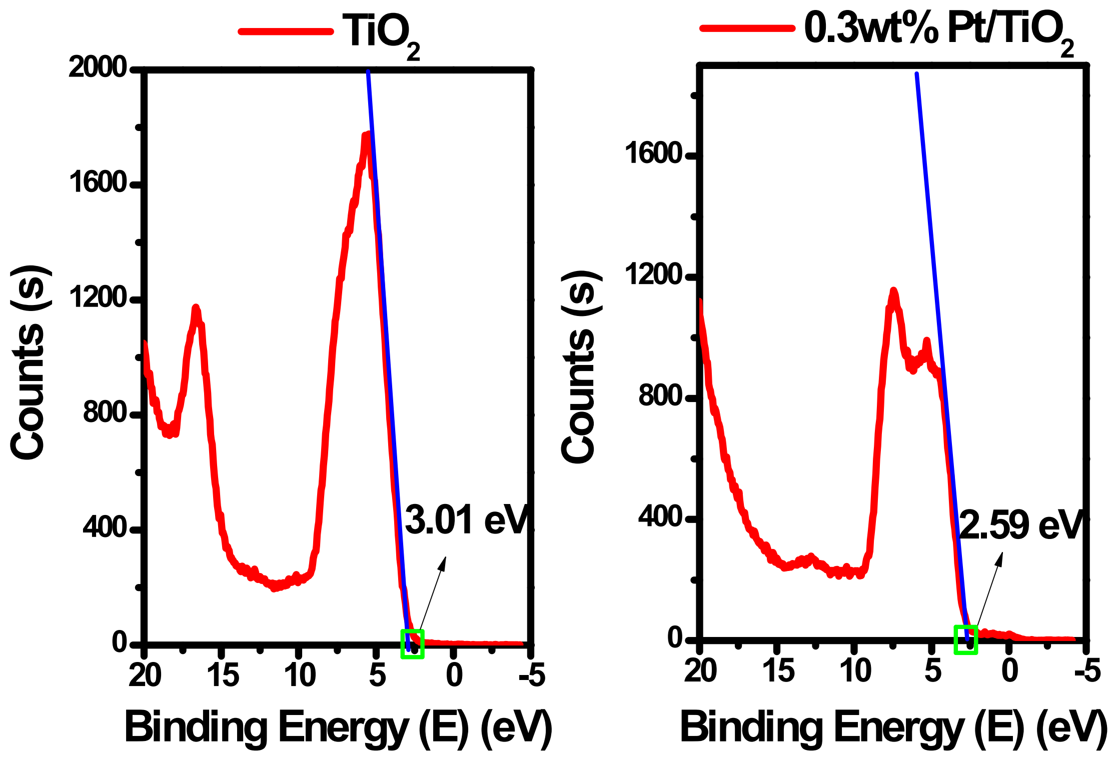

Figure S3 The XPS valence band scan spectrum of pure anatase TiO_2 NSs and 0.3 wt% Pt/TiO_2.

E. **The XPS survey scan and narrow scan analysis of Pt/TiO_2 nanocomposite**

The 0.3wt% Pt/TiO_2 was further characterized by XPS in Figure S4 (A-D) to illustrate their structural features and composition.

The XPS survey scan spectrum of 0.3wt% Pt/TiO_2 was shown in Figure S4 (A). The atomtic % of Pt, Ti and O of 0.3wt% Pt/TiO_2 was measured to be 0.05/33.88/66.07, and the wt % of Pt was measured to be 0.36 wt%, which was acceptable errors, indicating that Pt was deposited on the surface of TiO_2.

The XPS narrow scan spectra of Ti2p, Pt4f and O1s of the 0.3wt% Pt/TiO_2 was shown in Figure S4 (B-D). The Ti2$p_{1/2}$ and Ti2$p_{3/2}$ peaks were located at 464.6 and 458.8 eV assigned to TiO_2, respectively. The Pt 4$f_{5/2}$ and Pt 4$f_{7/2}$ peaks were located at 74.3 and 71.2 eV when Pt was loaded on TiO_2, which can be assigned to zero-valent Pt[1]. It proved that the Pt^{4+} was fully reduced to Pt^0.

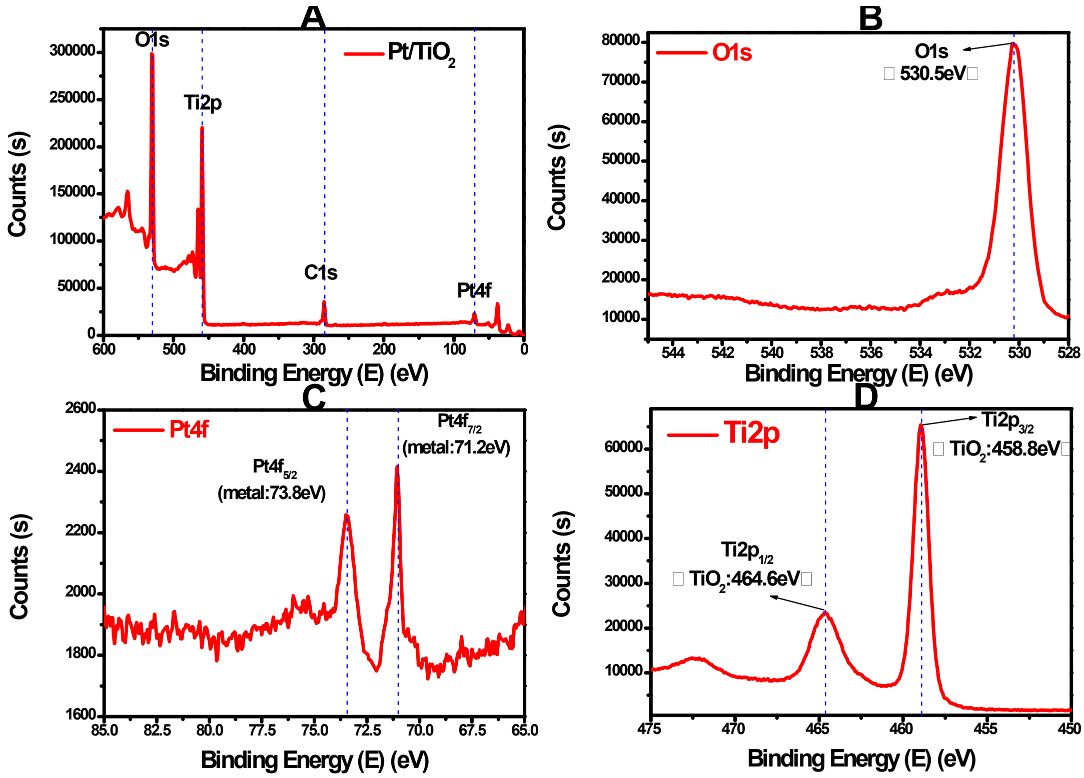

Figure S4 The XPS survey scan spectra of (A) Pt/TiO_2, the XPS narrow scans of (B) Ti2p, (C) W4f and (D) O1s of 0.3wt% Pt/TiO_2.

F. The ICP-AES analysis of Pt/TiO_2 nanocomposite

The content of Pt element for 0.3wt% Pt/TiO_2 nanocmposite was measured by ICP-AES and the Pt wt% was 0.34%. The weight ratio of Pt measured by ICP-AES was close to the theoretical value, which was consistent with the result of XPS, indicating that the synthesis of 0.3wt% Pt/TiO_2 photocatalyst was achieved.

Section 2. The detail information of characterization, photocatalytic test and analysis of intermediates of photocatalytic degradation of phenol

A. Characterization

The Powder X-ray diffraction (PXRD) analysis was carried out with a Bruker D8 Advance X-ray diffractometer using X radiation.

The scanning electron microscopy (SEM) image was taken on a JEOL JSM 6701F scanning electron microscopy operated at 30 kV. Oxford spectrometer Energy dispersive X-ray (EDX) spectroscopy being attached to SEM was used to observe and analyze the morphology of the samples, uniformity, element composition and distribution.

The particle sizes of samples were comparatively determined using Malvern Zetasizer Nano-ZS90 and Omecls-601A laser particle analyzers (Britain, Malvern Corporation).

X-ray photoelectron spectroscopy (XPS) was performed on an Escalab 250 Xi (monochromatic Al target) instrument for analysis of the surface elements and existing state, all the binding energy with carbon pollution C1s the binding energy of 284.8 eV for calibration.

UV-vis diffuse reflectance spectroscopy (DRS) of the products was recorded on a Cary 100 UV-vis-NIR spectrophotometer.

The ST2258C type multifunctional digital four point probe tester (China, Suzhou Jingge Electronic Co., LTD) was used to analysis of the volume resistivity and conductivity of samples.

The contents of the elements were determined by an inductively coupled plasma atomic absorption spectrometer (Perkin Elmer Optima model, 5300D). Operating conditions were: radio frequency incident power was 1400 W, flow rate of auxiliary argon was 0.2 L/min, flow rate of nebulizer argon was 0.9 L/min, flow rate of plasma gas was 18 L/min, flow rate of sample uptake was 1.5 mL/min, viewing mode was Axial, spray chamber type was Cyclonic, sample propulsion was peristaltic pump and three channel, torch type was fassel type and detector was segmented-array charge-couple.

The Fluorescence (FL) spectra of the as-fabricated products were detected with a Hitachi F-4500 fluorescence spectrophotometer with the Fluorescence intensity at 425 nm of 2-hydroxyterephthalic acid excited by 315 nm ±11 nm light, and the wavelength range was 400-600 nm.

B. Photocatalytic test

The phenol photocatalytic degradation over $Ti_{0.7}W_{0.3}O_2/TiO_2$ NCI was carried out by a photochemical reactor (BL-GHX-V, China) with a solar light-simulating source (BL-GGZ High pressure mercury lamp 150W). A schematic diagram of the photochemical reactor was shown in Figure S5. The concentration of phenol was 95 ppm, 0.45 g photocatalyst was added into a quartz tube and mixed with 1000 mL of phenol solution in each group. Prior to irradiation, the solution was magnetically stirred in the dark for 30 min to establish an adsorption and desorption equilibrium. The solutions were fetched, centrifuged and filtered through regular time intervals. The total reaction time was 360 min under the pH of 4.5.

The UV-Vis spectrum and concentration of phenol taken out from the reaction solution at given intervals of illumination were obtained with a Lambda 35 spectrometer (Perkin Elmer, USA).

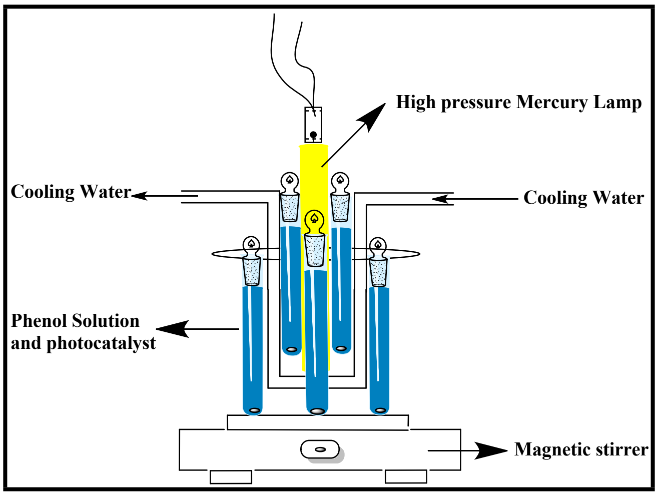

Figure S5 Schematic diagram of the photochemical reactor with an internal light source.

C. Analysis of Intermediates of Phenol Photocatalytic Degradation

Photocatalytic degradation intermediates of phenol were determined with HPLC-MS (Waters ZQ 4000/2695, USA) and GC-MS (Agilent 6890N/5973, USA). The intermediate products were separated on a Waters ZQ 4000/2695 HPLC-MS system, which is comprised of a Waters 2996 Photodiode Array Detector and electrospray ionization (ESI) source. There into, the Waters 2695 separation unit includes quaternary pumping system, the vacuum degasification machine, auto injector and column oven. The chromatograms were recorded on a personal computer with MasslynxV4.1 software (Waters, USA). All chromatographic experiments were carried out at ambient temperature with C18 column from Diamonsil (150 x 4.6 mm). The UV absorbance

detection was performed at 190-300 nm. A volume of 20 µL was injected for separation in a reverse phase mode and mobile phase of ultra-pure water/acetonitrile (65/35) with at a flow rate of 0.4 mL/min. The mass spectrometer was operated in positive mode and negative mode. The main parameters of MS were as follows: the m/z was 50-500 amu, source temperature was 110 ℃, desolvation temperature was 350 ℃, capillary was 3.00 kV, cone was 30 V and collision gas was nitrogen.

The capillary column used of GC-MS was a Agilent 19091J-413 HP-5 (methylsiloxane, 30 m x 0.32 mm I.D., 0.25 µm-thick film) fitted with a supelco guard column (deactivated methylsiloxane, 1 m x 0.32 mm I.D.). The operating conditions were: injection port temperature, 280 ºC; interface temperature, 280 ºC; column oven temperature, 60 ºC for 2 min, ramped at 8ºC min^{-1} to 280 ºC with a 15 min hold; helium carrier gas (flow rate of 3.4 mL min^{-1} at 50 ºC); 0.5-1 µL injection volume. The split/splitless injector was operated in the Split mode (Split ratio: 0.1:1, Split flow: 0.1 mL min^{-1}, Total flow: 3.4 mL min^{-1}) for 2 min after injection of the sample. Regular replacement of the injection port liner and guard column was found to be necessary for this procedure because of the presence of large amounts of in volatile, possibly high-molecular-weight, compounds in the analyzed samples.

Section 3. Particle Size Analysis

The average particle size and a distribution of anatase TiO_2 NSs and $Ti_{0.7}W_{0.3}O_2$ NPs were obtained using a laser particle size analyzer and showed in Figure S6.

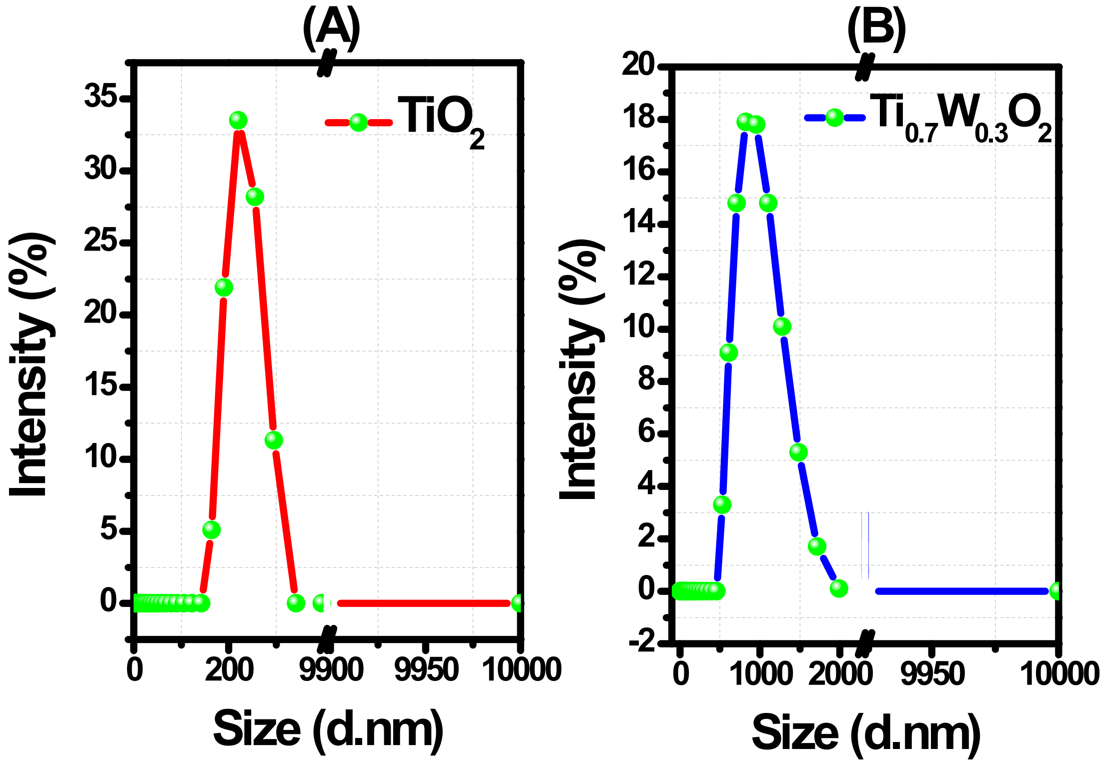

Figure S6 Diameter distribution histograms of the as-fabricated samples: (A) TiO_2 NNs; (B) $Ti_{0.7}W_{0.3}O_2$ NPs.

Section 4. The Ultraviolet Photoemission Spectroscopy (UPS) analysis and the energy level diagram

The UPS result of TiO_2 NSs and 5 wt% $Ti_{0.7}W_{0.3}O_2/TiO_2$ NCI was shown in Figure S7.

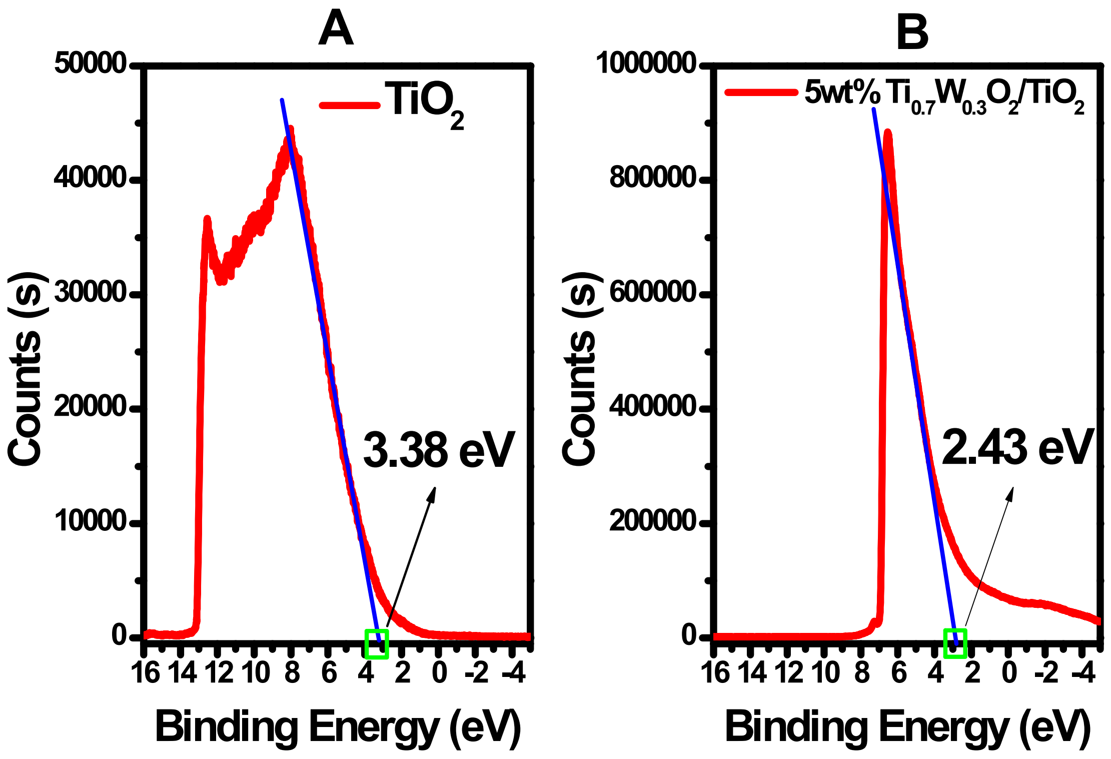

Figure S7 The ultraviolet photoemission spectroscopy (UPS) of (A) pure anatase TiO_2 NSs and (B) 5 wt% $Ti_{0.7}W_{0.3}O_2/TiO_2$ NCI.

The energy level diagram of $Ti_{0.7}W_{0.3}O_2/TiO_2$ NCI was showed in Figure S8.

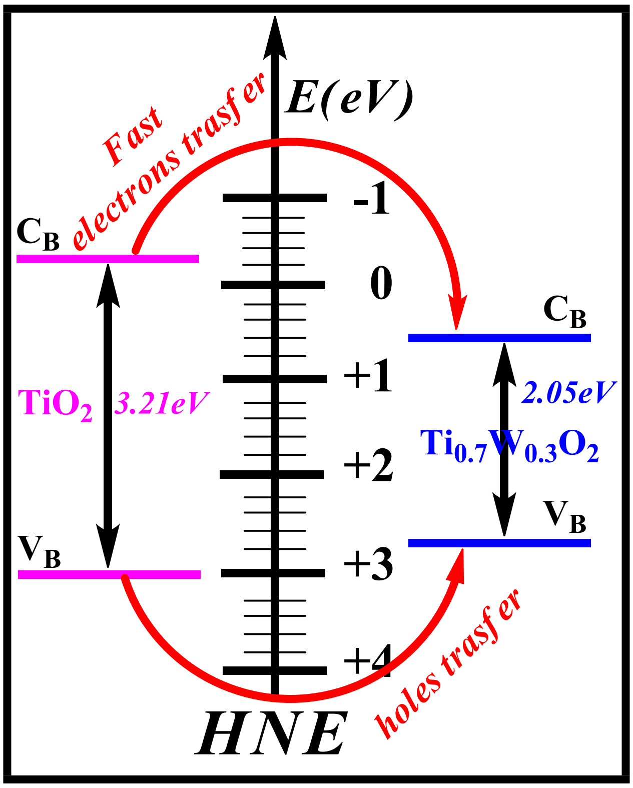

Figure S8 The energy level diagram of $Ti_{0.7}W_{0.3}O_2/TiO_2$ NCI.

Section 5. The UV-Vis spectra of phenol degradation catalyzed by P-25, Pt/TiO_2 and $Ti_{0.7}W_{0.3}O_2/TiO_2$ NCI

The UV-Vis spectra of phenol degradation catalyzed by P-25, Pt/TiO_2 and $Ti_{0.7}W_{0.3}O_2/TiO_2$ NCI were shown in Figure S9. The characteristic absorption bands at 270 nm of phenol diminished gradually as exposure time extended, indicating gradual phenol degradation in wastewater. What was more, the reduced speeds over different photocatalysts were inconsistent. The $Ti_{0.7}W_{0.3}O_2/TiO_2$ and Pt/TiO_2 exhibited much higher photocatalytic activity than that of P-25, which indicated that the photocatalytic activity of TiO_2 NSs could be greatly improved by melioration of $Ti_{0.7}W_{0.3}O_2$ and Pt NPs. Meanwhile, the photocatalytic degradation of phenol over different $Ti_{0.7}W_{0.3}O_2/TiO_2$ NCI was also different. The characteristic absorption bands at 270 nm of phenol scarcely declined over pure rutile $Ti_{0.7}W_{0.3}O_2$ NPs as the exposure time extended, indicating that there was no phenol photocatalytic performance. This may be similar to Pt NPs, which can be only served as co-catalysts.

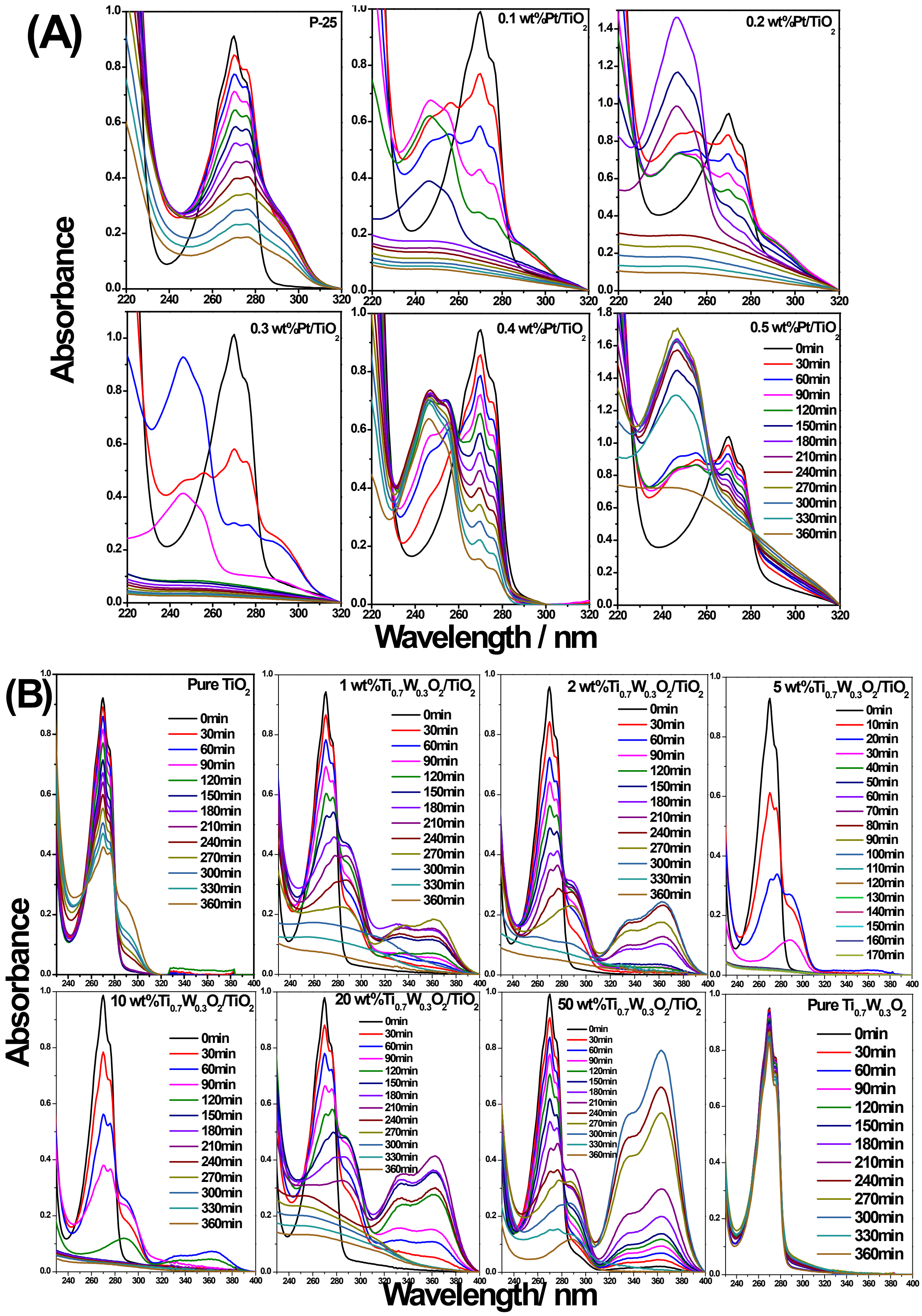

Figure S 9 The UV-Vis spectra of phenol degradation catalyzed by (A) P-25, Pt/TiO_2 and (B) $Ti_{0.7}W_{0.3}O_2/TiO_2$.

Simultaneously, the characteristic absorption bands at 270 nm of phenol exhibited bathochromic shifts to some extent concomitantly with intermediates such as catechol and hydroquinone[2]. It was worthwhile to mention that there were several new absorption bands at 333 nm and 363 nm indicating the formation of other intermediates. Then, all the absorption

bands disappeared, indicating the phenol photodegradation was complete finally. It was easily to find that the intensity of absorption bands at 333 and 363 nm presented certain regularity with different loaded ratio of $Ti_{0.7}W_{0.3}O_2$ NPs. The teeny intensity of the two absorption bands for 5 wt% $Ti_{0.7}W_{0.3}O_2/TiO_2$ NCI was the lowest, and more or less $Ti_{0.7}W_{0.3}O_2$ NPs loaded ratio would increase the intensity immensely. It indicated that intermediates for 5 wt% $Ti_{0.7}W_{0.3}O_2/TiO_2$ with the highest photocatalytic activity were decomposed rapidly rather than less production, and 5 wt% was the most optimal loaded level for $Ti_{0.7}W_{0.3}O_2/TiO_2$ NCI clearly.

Section 6. Compared the characteristic UV-Vis spectra of phenol degradation intermediates

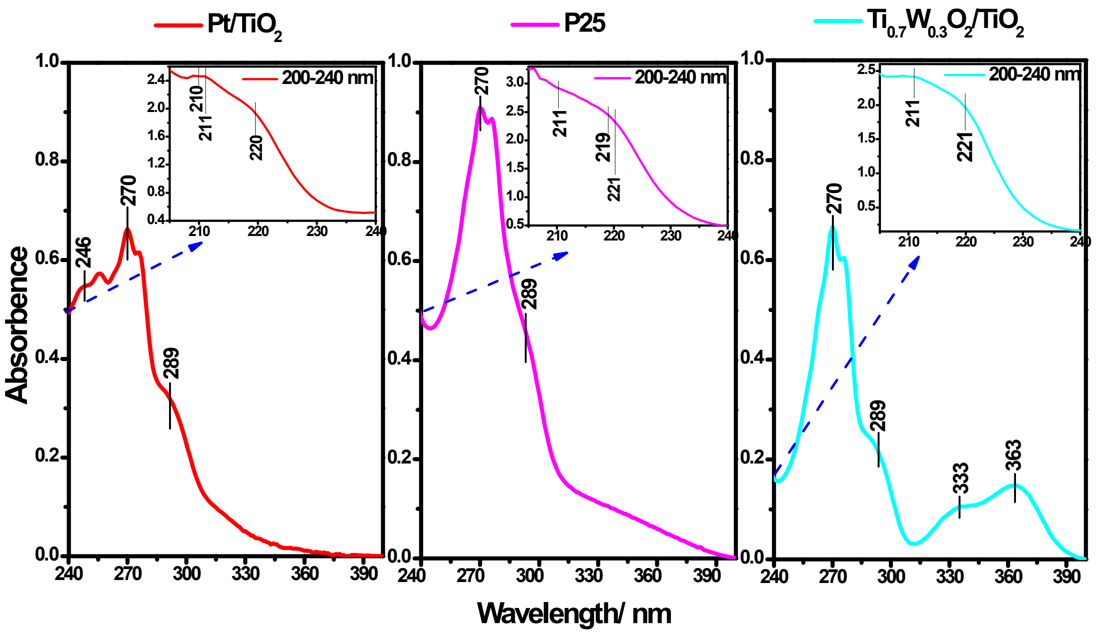

Figure S10 Compared the characteristic UV–Vis spectra of phenol degradation intermediates for P-25, Pt/TiO_2 and $Ti_{0.7}W_{0.3}O_2/TiO_2$ NCI.

Section 7. The LC chromatogram, UV-Vis spectrogram and Mass spectrum of main intermediates.

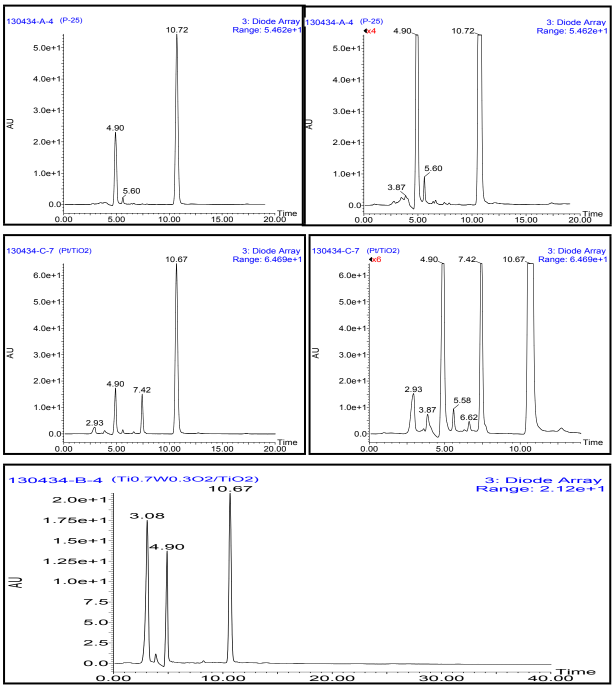

Figure S11-a The LC chromatogram of phenol photocatalytic degradation aqueous solution of P-25, Pt/TiO_2 and $Ti_{0.7}W_{0.3}O_2/TiO_2$ NCI, respectively.

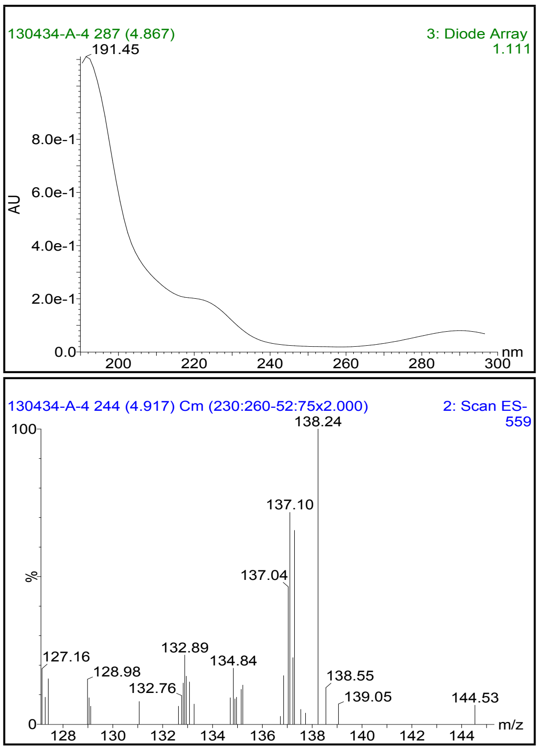

Figure S11-b The UV spectrogram and mass spectrum for the intermediate A1 (t_R=4.90 min, λ=289.45; 221.45; 191.45, $M/Z_{(Es-)}$=137.10, M/Z=138.00) of phenol photocatalytic degradation over P-25.

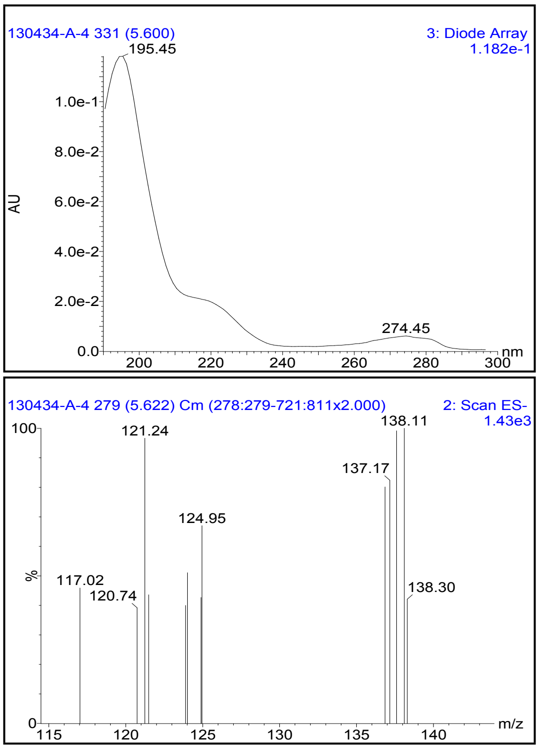

Figure S11-c The UV spectrogram and mass spectrum for the intermediate B1(t_R=5.60 min, λ=274.45; 219.45; 195.45, M/Z =121.24, M/Z=122.00) of phenol photocatalytic degradation over P-25.

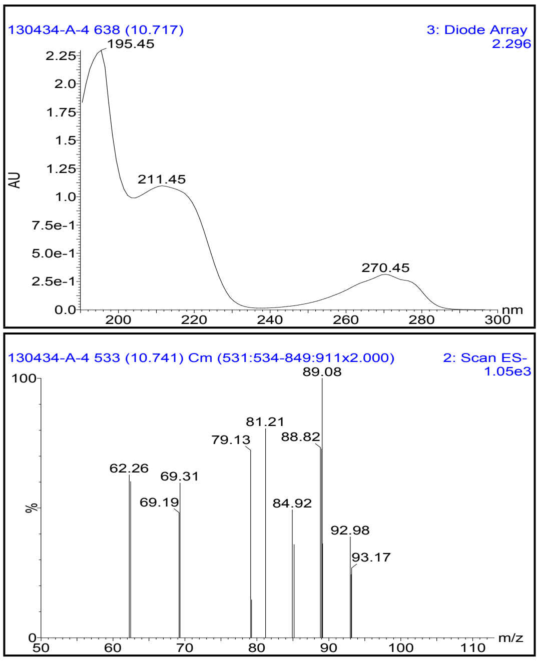

Figure S11-d The UV spectrogram and mass spectrum for the substance C1 (t_R=10.72 min, λ=270.45; 211.45; 195.45, $M/Z_{(Es-)}$ =92.98, M/Z=94.00) of phenol photocatalytic degradation over P-25.

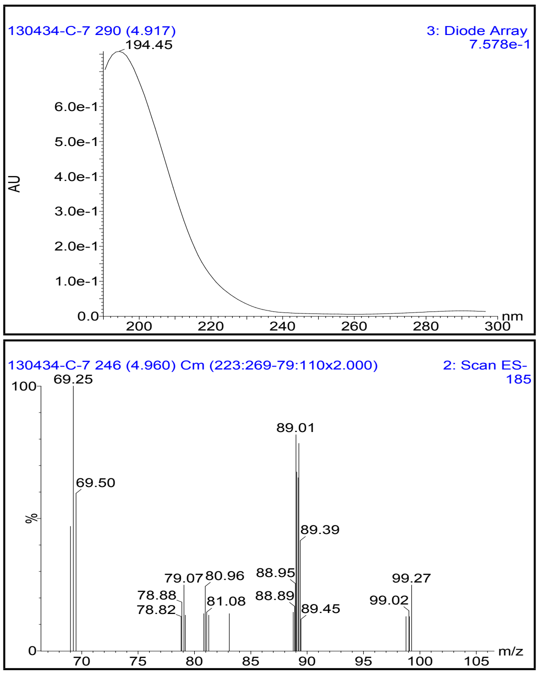

Figure S11-e The UV spectrogram and mass spectrum for the substance A2 (t_R=4.90 min, λ=194.15, $M/Z_{(Es-)}$=89.01, M/Z=90.00) of phenol photocatalytic degradation over Pt/TiO_2.

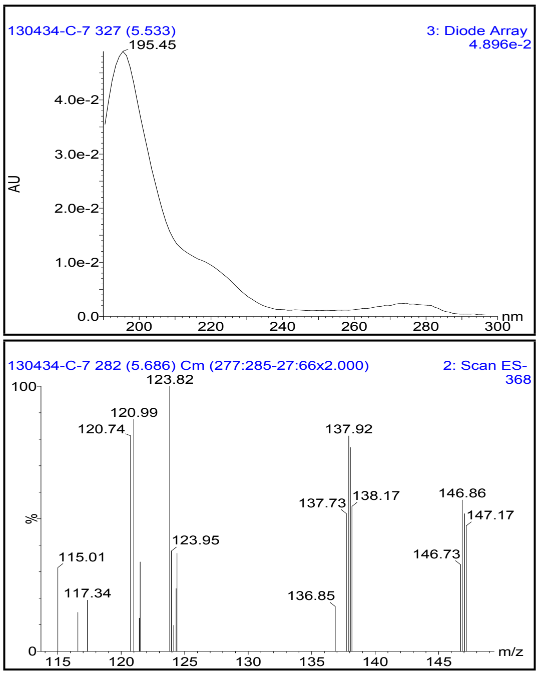

Figure S11-f The UV spectrogram and mass spectrum for the substance B2 (t_R=5.58 min, λ=274.45; 219.45; 195.45, $M/Z_{(Es-)}$=120.99, M/Z=122.00) of phenol photocatalytic degradation over Pt/TiO_2.

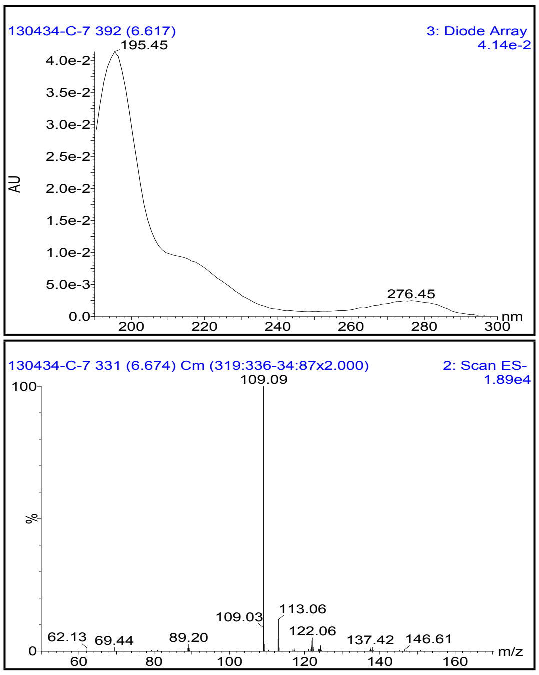

Figure S11-g The UV spectrogram and mass spectrum for the substance C2 (t_R=6.62 min, λ=276.45; 214.45; 195.45, $M/Z_{(Es-)}$=109.09, M/Z=110.00) of phenol photocatalytic degradation over Pt/TiO_2

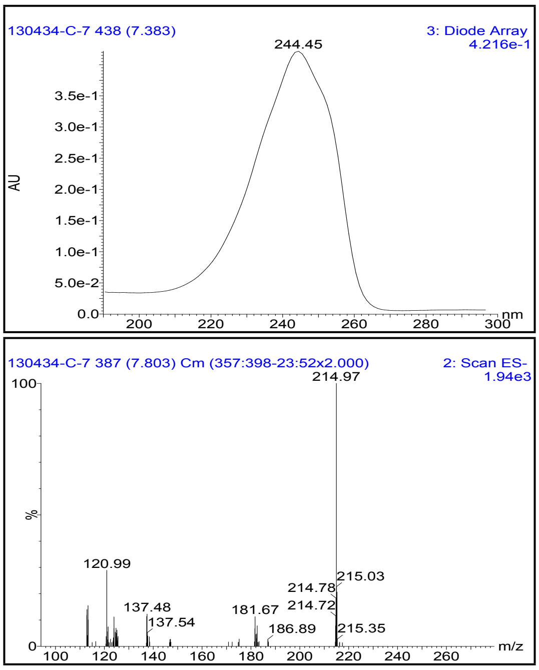

Figure S11-h The UV spectrogram and mass spectrum for the substance D2 (t_R=7.42 min, λ=244.45, $M/Z_{(Es-)}$=214.97, M/Z=216.00) of phenol photocatalytic degradation over Pt/TiO_2.

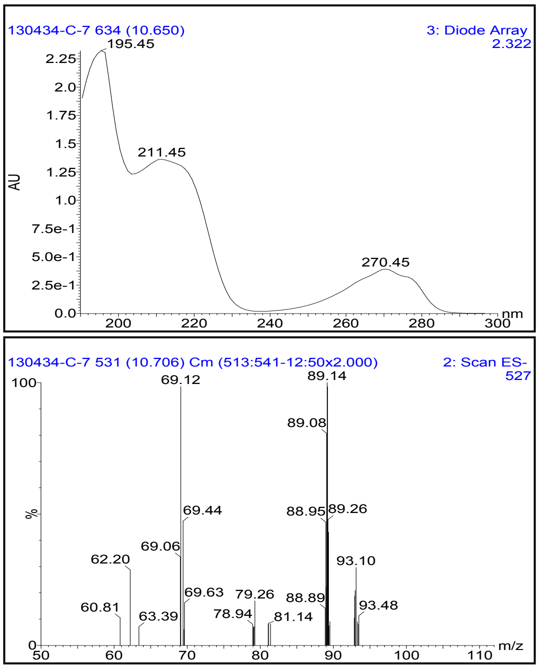

Figure S11-i The UV spectrogram and mass spectrum for the substance E2 (t_R=10.67 min, λ=270.45; 211.45; 195.45, $M/Z_{(Es-)}$ =93.10, M/Z=94.00)of phenol photocatalytic degradation over Pt/TiO_2.

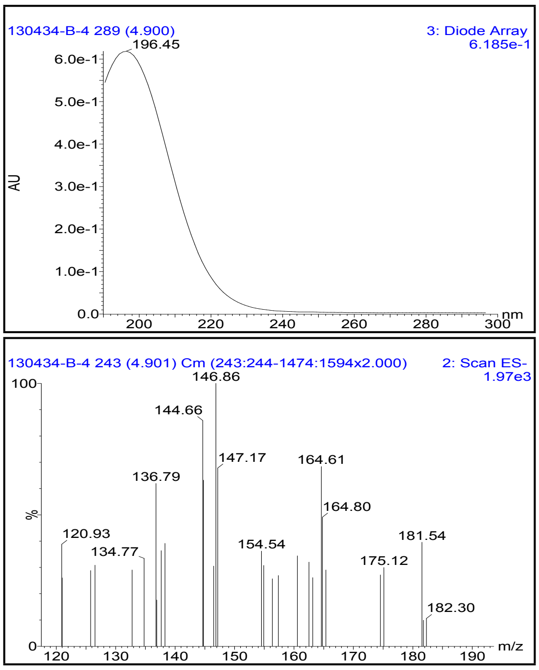

Figure S11-j The UV spectrogram and mass spectrum for the substance A3 (t_R=4.90 min, λ=196.15 nm, $M/Z_{(Es-)}$=164.61, M/Z=166.00) of phenol photocatalytic degradation over $Ti_{0.7}W_{0.3}O_2/TiO_2$ NCI.

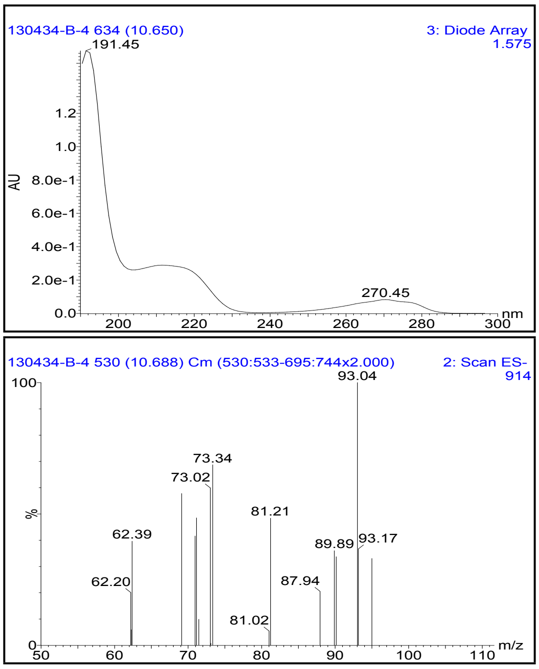

Figure S11-k The UV spectrogram and mass spectrum for the substance B3 (t_R=10.67 min, λ=270.45; 211.45; 195.45, M/Z(Es-) =93.04, M/Z=94.00) of phenol photocatalytic degradation over $Ti_{0.7}W_{0.3}O_2/TiO_2$ NCI.

Section 8. The UV-Vis absorption spectra, GC chromatogram and Mass spectrum of GC-MS for intermediates.

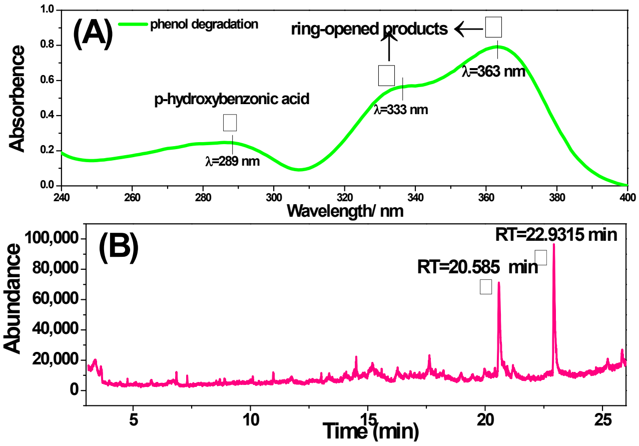

Figure S 12 (A) The characteristic UV-Vis absorption spectra of phenol photocatalytic degradation intermediates after 330 min irradiation over 50 *w* % $Ti_{0.7}W_{0.3}O_2/TiO_2NCI$, (B) the GC chromatogram of phenol photocatalyitc degradation intermediates after 330 min irradiation.

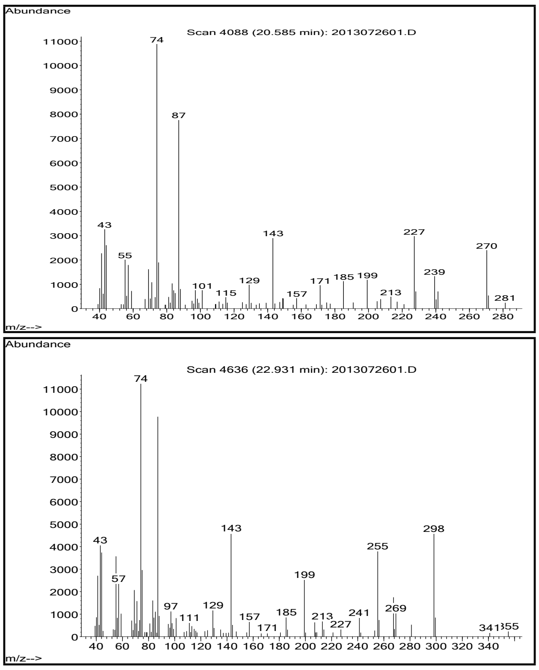

Figure S13 The mass spectrum of GC-MS for intermediates of phenol photocatalytic degradation over $Ti_{0.7}W_{0.3}O_2/TiO_2$ NCI: ① RT= 20.585 min (m/z=270) and ② RT= 22.9315 min (m/z=298).

Section 9. The analysis result and possible structure of each intermediate.

The result and possible structure of each intermediate were shown in Table S1 and Figure S13.

Table S1 The result and possible structure of each phenol photocatalytic degradation intermediates over P-25, Pt/TiO_2 and $Ti_{0.7}W_{0.3}O_2/TiO_2$ NCI

Samples	M/Z	Structure
P-25	94.0	A
	110.0	B
	110.0	C
	110.0	D
	122.0	E
	138.0	F
Pt/TiO_2	90.0	G
	94.0	A
	110.0	B
	110.0	C
	110.0	D
	122.0	E
	216.0	H
$Ti_{0.7}W_{0.3}O_2/TiO_2$	94.0	A
	110.0	B
	110.0	C
	110.0	D
	166.0	I
	270.0	J
	298.0	K

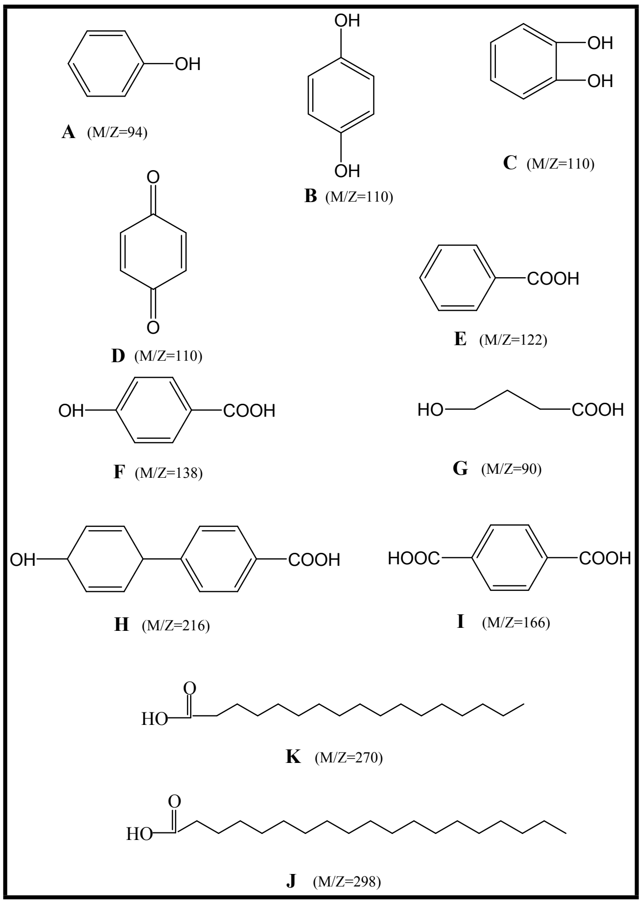

Figure S14 The possible structure of intermediates for phenol photocatalytic degradation over P-25, Pt/TiO_2 and $Ti_{0.7}W_{0.3}O_2/TiO_2$ NCI.

Notes and references

1. G. Q. Guo, F. Qin, D. Yang, C. C. Wang, H. L. Xu and S. Yang, *Chem. Mater*. 2008, 20, 2291-2297.
2. L. Y. Xiong, L. Z. Zheng, J. P. Xu, D. Zheng, J. H. Li, X. J. Li, J. Sun, Q. Liu, L. N. Niu, S. M. Yang and J. Xia, *Environ. Chem. Lett.*, 2010, 39, 1201-1208.
